# Towards a Molecular Understanding of Cation‐Anion Interactions and Self‐aggregation of Adeninate Salts in DMSO by NMR and UV Spectroscopy and Crystallography

**DOI:** 10.1002/cphc.202100098

**Published:** 2021-08-08

**Authors:** Dominique M. S. Buyens, Lynne A. Pilcher, Emil Roduner

**Affiliations:** ^1^ Institute of Physical Chemistry University of Stuttgart 70569 Stuttgart Germany; ^2^ Department of Chemistry University of Pretoria Pretoria 0002 Republic of South Africa

**Keywords:** nucleobases, aggregation, hydrogen bonding, stacking interactions, ion-pairing

## Abstract

Rare anionic forms of nucleic acids play a significant biological role and lead to spontaneous mutations and replication and translational errors. There is a lack of information surrounding the stability and reactivity of these forms. Ion pairs of mono‐sodium and ‐potassium salts of adenine exist in DMSO solution with possible cation coordination sites at the N1, N7 and N9 atoms of the purine ring. At increasing concentrations π‐π stacked dimers are the predominant species of aggregates followed by higher order aggregation governed by coordination to metal cations in which the type of counter ion present has a central role in the aggregate formation.

## Graphical abstract

**Adeninate‐metal ion pairs exist in DMSO** solution whereby the metal ion coordinates via the N3, N7 or N9 nitrogen atom or chelated between the N3–N9 atoms of the purine ring. The monovalent metal ion governs higher order aggregate formation in which the identity of the metal ion plays a role in structural arrangement. These results provide insight into the reactivity and stabilization of anionic adenine in the presence of important biological ions.

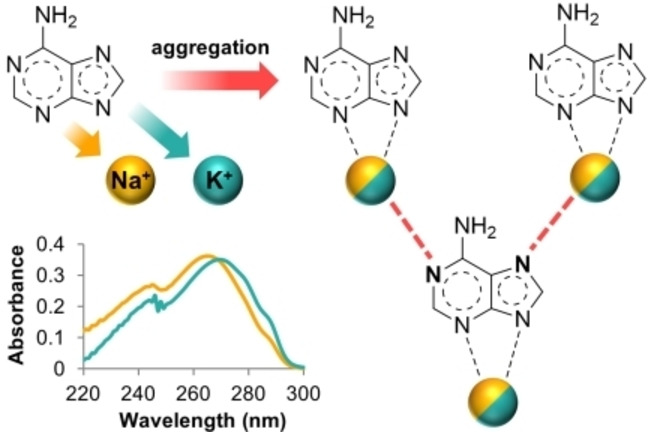

## 1 Introduction

Non‐covalent interactions governing the aggregation of isolated purine bases, such as hydrogen bonding and π‐π stacking, have been investigated in order to gain greater understanding of their role in structure, function and stability since Watson and Crick (1953) proposed the structure of DNA. It is well known that adenine is involved in Watson‐Crick^[1]^ (WC) along with reversed WC,^[2]^ Hoogsteen^[3]^ (HG) and reversed HG^[4]^ type hydrogen bonding with thymine and uracil in DNA and RNA, respectively. The self‐[^5^, ^6^, ^7^] and hetero‐association of adenine and adenine derivatives with other nucleobases[^8^, ^9^] and carboxylic acids[^10^, ^11^, ^12^] has been extensively investigated.

The binding of alkali metal ions such as Na^+^ and K^+^ to nucleobases influences the structural integrity of DNA and play a role in synthesis, replication, recognition and cleavage of DNA and RNA.[^13^, ^14^] Binding of metal ions can interfere with hydrogen bonding and stacking interactions between base pairs and compromise the structural stability of DNA.^[15]^ Experimentally, the affinity for alkali metals increase in the order guanine>cytosine>adenine>thymine>uracil, following the trend of proton affinity in nucleobases.[^13^, ^16^] The preferred binding sites of metal ions are the N7 in adenine, the N7 and O6 atoms in guanine, the N3 and O2 atoms in cytosine and the O4 in thymine and uracil.^[13]^ The three dimensional folding adopted by nucleic acid repeat sequences show ion dependency[^17^, ^18^] which further highlights the role and importance of monovalent metal ions in DNA structure.

The multi‐site coordination and ability of nucleobases to interact non‐covalently has rendered them desirable ligands in the synthesis of stable bio‐metal‐organic frameworks that can have structural flexibility.[^19^, ^20^, ^21^, ^22^] Metal complexes of adenine can have mono‐ or bidentate coordination through different combinations of N3, N7 and N9 nitrogen atoms and its deprotonated form (adeninate anion) can be di, tri‐ or tetradentate, have varying coordinate combinations through the N1, N3, N7 and N9 atoms (see reference within the review^[20]^ and reference^[23]^) and bridging through the N3, N7 and N9 atoms.^[24]^

The presence of disfavored tautomeric and anionic forms of nucleic acids can form WC‐like mispairs (i. e. guanine‐thymine, adenine‐cytosine and guanine‐uracil) that are involved in mutations and replication and translational errors.[^25^, ^26^, ^27^, ^28^] These forms of nucleic acids are also believed to play a role in nucleic acid catalysis^[29]^ and RNA‐ligand recognition.^[30]^ The anion forms, such as adeninate anion, features in investigations surrounding the photostability of nucleic acids in DNA[^31^, ^32^] and is also used in the synthesis of N9‐substituted adenines, which have important biological roles such as anti‐asthmatic^[33]^ and anti‐inflammatory activity,^[34]^ activity against diseases such as multiple sclerosis and autoimmune disease.^[35]^

There is a lack of information surrounding anionic forms of nucleobases and is a topic being addressed currently.[^26^, ^36^] Studies involving the chemical properties of the free adeninate anion and its interactions with monovalent alkali metals are limited. In this paper the ion‐pair formation of the adeninate anion with the biologically important monovalent metals, Na^+^ (Na‐Ade) and K^+^ (K‐Ade), and the self‐aggregation of these ionic complexes in DMSO are investigated using NMR and UV spectroscopy as well as XRD of crystal structures. In addition, we have studied the influence of the 15‐crown‐5 (15C5) and 18‐crown‐6 (18C6) ether on the spectroscopic and crystal structures of Na‐Ade and K‐Ade. To this end we show that ion‐pair formation exists between the adeninate anion and the counter ions even at extremely dilute concentrations and that self‐aggregation of these complexes is highly influenced by the counter ion present. Furthermore, there is a possibility of complex formation between K‐Ade and the 18‐crown‐6 ether.

## 2 Results and Discussion

### 2.1 Ion Pairing

The use of organic solvents such as DMSO, DMF, methanol etc., has an effect on the non‐covalent interactions (such as hydrogen bonding, pi‐pi stacking and cation binding) of nucleic acids^[37]^ and therefore on the interactions of nucleobases as well. However, many thermodynamic properties of nucleobases are reported in organic solvents or co‐solvents of water and organic solvents in order to enhance the chemical property being studied or to understand the effect of solvent.^[38]^ It is important to note that often nucleobase derivatives are used in aqueous media due to the low solubility of free nucleobases.^[39]^

In order to gain an understanding of the interactions between adenine and the counter ion, the experiments are conducted in a solvent which allowed for the solubility of adenine and the adeninate salt and allows for potential ion‐pairing. DMSO, which acts as a hydrogen bond acceptor only, will not form hydrogen bonds at nitrogen sites of adenine rendering these sites available for metal ion coordination. Moreover, further studies conducted of the regio‐selectivity of the adeninate salt (publication in preparation) require the use of DMSO and hence, to maintain a common solvent, DMSO is used to perform these studies.

**Figure 1:**
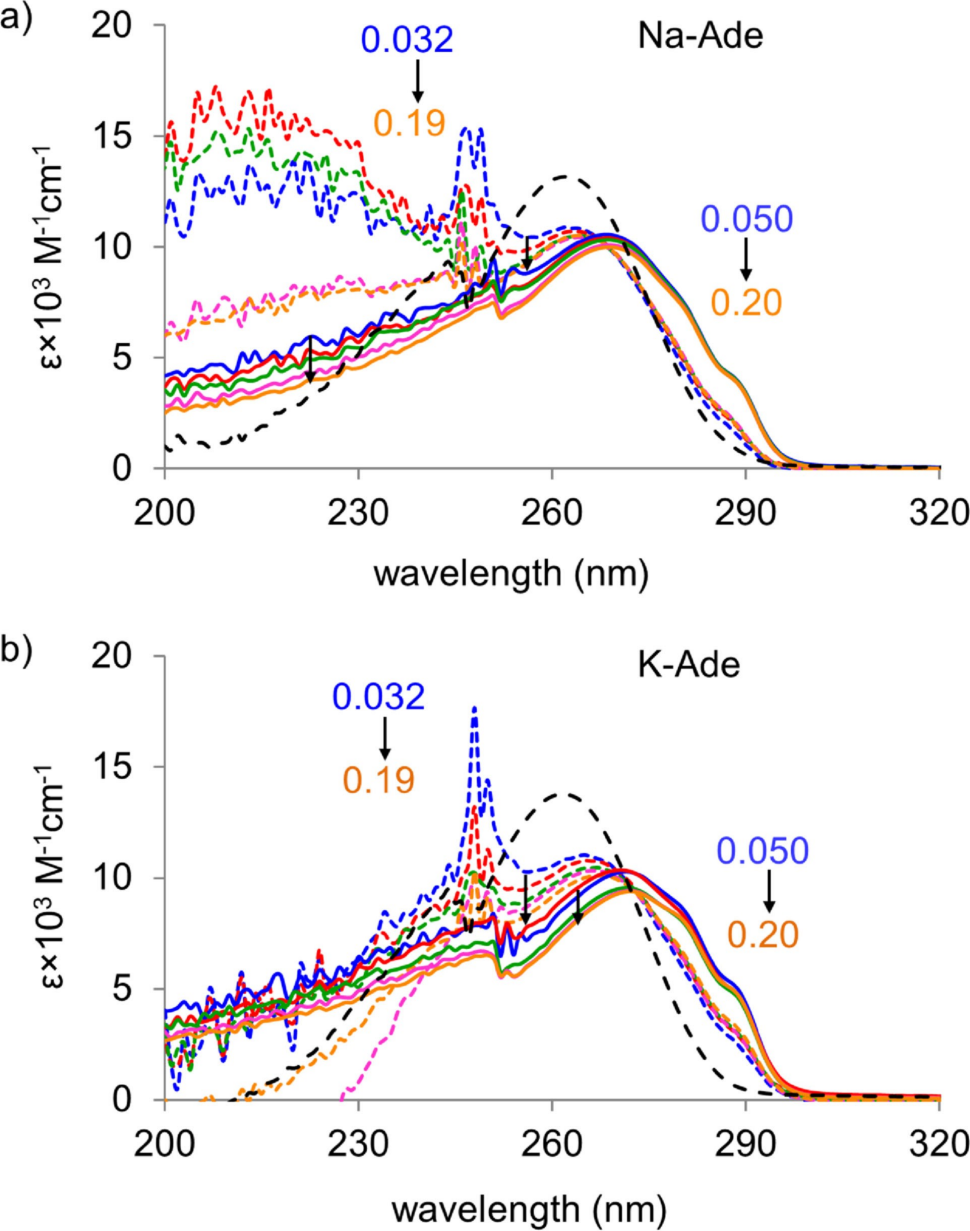
Concentration dependent UV absorption profile of a) Na‐Ade and b) K‐Ade in DMSO for the concentration range (0.05 – blue, 0.075 – red, 0.15 – green, 0.10 – pink, 0.20 – orange, in mM) and in the presence of a large excess of 15C5 (126 eq) and 18C6 (117 eq) ether, respectively (dashed black lines).

The UV studies performed showed a significant dependence on the counter ion present indicating that ion pairs exist between the adeninate anion and the Na^+^/K^+^ counter ion. The normalized UV absorption profiles of Na‐Ade and K‐Ade in anhydrous DMSO are shown in Figure 1 (UV absorbance spectra, Figure S21). The λ_max_ and shoulder bands observed are reported in Table 1. The blueshift of the λ_max_ from K‐Ade (272 nm) to Na‐Ade (269 nm) in DMSO is associated with the larger stabilization of the ground state of the anion by the Na^+^ counter ion which has a stronger cationic field strength due to its smaller ionic radius compared to K^+^.[^40^, ^41^]

**Table 1:** The λ_max_ and (shoulder) bands in the UV absorption spectrum of Na‐ and K‐Ade, Na‐Ade(15C5) and K‐Ade(18C6) in DMSO/H_2_O and DMSO.

Solvent	λ_max_ [nm]
DMSO/H_2_O+NaH	246, 250, 264→265 (278, 287)
DMSO/H_2_O+KH	248, 250, 264→269 (278, 287)
DMSO+NaH	251, 254, 269 (278, 287)
DMSO+KH	251, 253, 272 (278, 287)
DMSO/H_2_O + NaH+15C5	245, 262
DMSO/H_2_O+KH+18C6	245, 262

Water molecules should facilitate the dissociation of the counter ion from the adeninate ion pair. Studying the system with a small amount of water present (5.2 % v/v H_2_O) provides insight into i) how the Na‐ and K‐Ade complexes respond to interactions with water and ii) how the M‐Ade concentration dependent characteristics of the bands change (normalized UV absorption profiles in DMSO/H_2_O, Figure S22). The blueshift from DMSO to DMSO/H_2_O for both ionic complexes is due to the hydrogen bonding of water stabilizing the ground state of the anion. Conversely, the solvation of the adeninate anion by polar solvents stabilizes the π* orbital, resulting in a redshift of the λ_max_ with increasing polarity and hydrogen‐acceptor properties of the solvent.^[42]^ A M‐Ade concentration dependent redshift is observed in DMSO/H_2_O, having the Na‐Ade shift from 262 to 265 nm and K‐Ade shift from 264 to 269 nm, accompanied by the increase in shoulder band intensity at 278 and 287 nm, which are attributed to the presence of the cationic and anionic species.^[42]^ These shoulder bands decrease in intensity as the concentration of Na‐ and K‐Ade decreases as the presence of water molecules assists the dissociation of ion‐pairs. In DMSO, the concentration‐dependent redshift of the band around 265 nm for Na‐ and K‐Ade is absent and the band intensity at 278 and 287 nm is larger in magnitude than in DMSO/H_2_O, indicating the ion‐pairs are the predominate species.

The UV spectroscopy study of Na^+^ and K^+^ ion complexation to the adeninate anion is conducted alongside the studies in the presence of 15C5 and 18C6 (Figure 1, dashed), respectively, in order to confirm the presence of ion‐pairs. In the presence of the large excess of 15C5 and 18C6, the shoulder profile is absent and the λ_max_ blueshifts to 262 nm for both Na‐ and K‐Ade as the free adeninate anion is formed, thus confirming that ion‐pairs exist in the absence of the crown ethers. Narrow finger like bands around the 245–250 nm region in the DMSO/H_2_O spectra are ascribed to DMSO‐water‐metal counter ion complexes as they also appear in the cation‐DMSO solutions in the absence of the adeninate anion (SI Figure S23). In the Na‐Ade DMSO/H_2_O UV spectra, a broad band lying below 235 nm is observed and is largely suppressed in DMSO and in the presence of 15C5 and is attributed to the population of higher lying bright ^1^ππ* state(s) of the adeninate anion by the excitation at 220 nm.^[32]^

### 2.2 Coordination Sites of Na^+^ and K^+^ with Adeninate Anion

The addition of base, NaH/KH, to adenine in DMSO‐d_6_ results in deprotonation, shielding all the purine protons in NMR relative to the peaks of adenine as electron density is gained in the purine ring, Table 2. The Na^+^ counter ion, with the larger charge/radius ratio, has stronger ion and ion‐induced dipole interactions with the adeninate anion, and this is reflected in the larger deshielding of the C2‐H and C8‐H protons relative to K‐Ade (Δδ of 0.11 and 0.17 ppm at dilute and 0.18 and 0.24 at saturated concentrations). The larger shielding of the C8‐H is ascribed to the ring current effect (Figure S5).

**Table 2:** ^1^H and ^13^C NMR chemical shifts (ppm) of adenine, Na‐Ade, K‐Ade and K‐Ade(18C6) in DMSO‐d_6_ as solvent.

	^1^H NMR chemical shifts				
	C2‐H	C8‐H	NH_2_		
Adenine^[a]^	8.11	8.09	7.08		
Na‐Ade^[b]^	7.90	7.66	5.94		
K‐Ade^[c]^	7.72	7.42			
K‐Ade(18C6)^[d]^	7.81	7.50

	^13^C NMR chemical shifts				
	C2	C4	C5	C6	C8
Adenine^[a]^	152.4	150.6	118.2	155.6	139.2
Na‐Ade^[b]^	146.9	157.7	119.7	154.3	149.4
K‐Ade^[c]^	147.9	160.7	121.7	155.0	150.5
K‐Ade(18C6)^[d]^	147.5	161.2	121.8	154.0	151.1

[a] Adenine at 73.2 mM [b] Na‐Ade at 52.2 mM, [c] K‐Ade at 84.8 mM [d] K‐Ade(18C6) at 78.7 mM.

The gained negative charge from deprotonation of adenine is shared on all five nitrogen atoms of the purine ring which in turn shields the neighboring carbons in the ^13^C NMR spectra, hence the deshielding of carbons indicates which neighboring nitrogen atoms are involved in the ionic bond. In Table 2, the ^13^C NMR chemical shifts for the imidazole ring carbons i. e. C4, C5 and C8, are deshielded relative to adenine, highlighting the 5 membered ring's involvement in the ionic bond. Moreover, the C4 and C8 are the most deshielded, suggesting the N9 as the site of coordination. The deshielding of the ^1^H and ^13^C NMR chemical shifts of K‐Ade(18C6) suggests that the 18C6 ether does not remove the K^+^ counter ion from K‐Ade.

**Figure 2:**
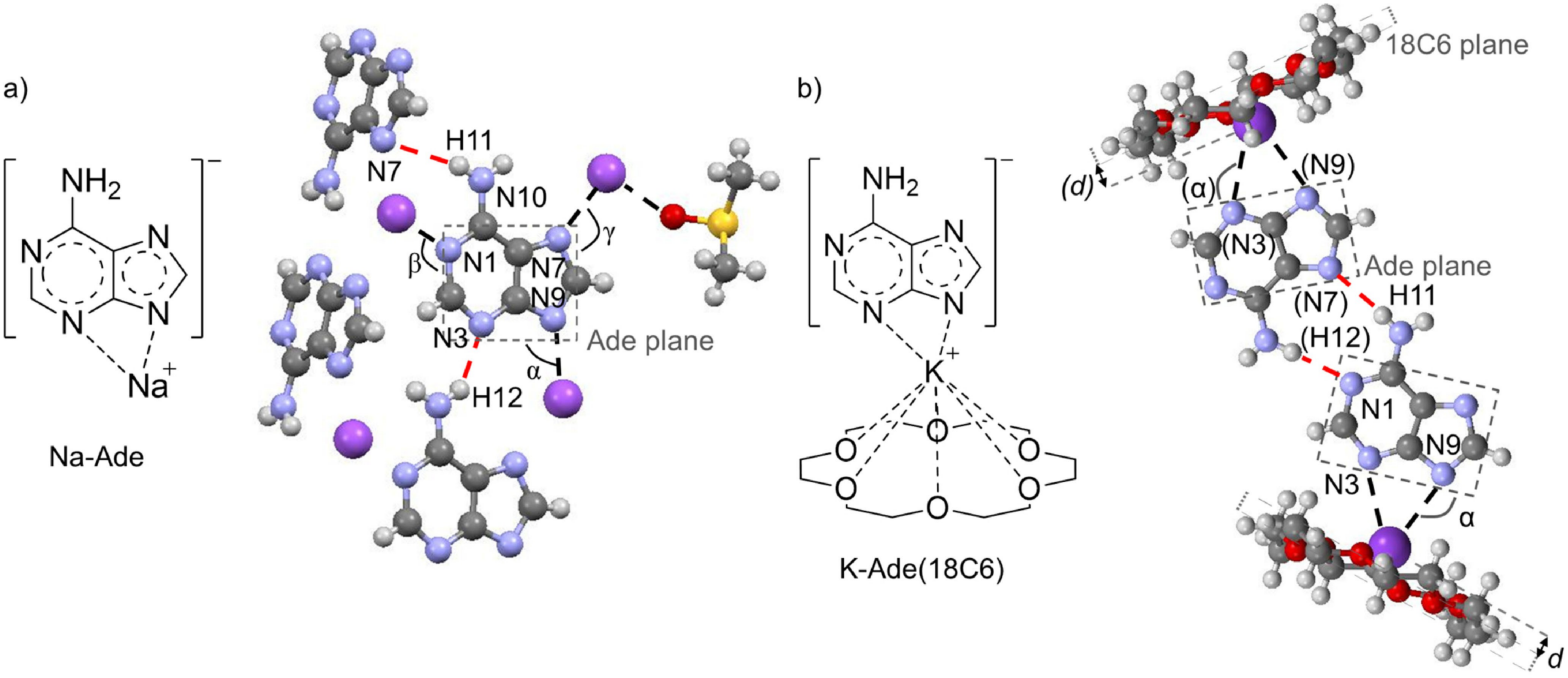
Molecular structure of a) Na‐Ade and the Na^+^ coordination obtained from single crystal XRD of Na‐Ade and b) K‐Ade(18C6) and the hydrogen bonding (red lines) and interaction with the K^+^ ion at the N3 and N9 atoms (black lines). The numberings of specified atoms and dihedral angles for geometric parameters in Table S4, are shown.

From ^13^C NMR spectroscopy, the site of coordination of the counter ion with the adeninate anion cannot be determined with certainty. The crystal structures obtained of Na‐Ade and K‐Ade provide insight into the potential coordination sites in solution. The crystallographic information is given in Table S3 and selected geometric parameters of the crystal structure of Na‐Ade are given in Table S4. The crystal system for both the Na‐Ade and K‐Ade display a zigzag packing morphology (Figure S16a and S17a) along the a axis in which there is a T‐shape edge to face arrangement. Along the c axis, the next row of units is inverted along the b axis, cancelling the dipole moment of the units and lowering the energy. In both the Na‐ and K‐Ade crystal structures, two DMSO‐d_6_ solvent molecules per Na‐Ade/K‐Ade are trapped within the channels formed by four Na‐Ade and K‐Ade (Figure S16b and S17b) units packed edge to face to form a rectangular cage. The DMSO‐d_6_ molecules occur in groups of two, oriented in the opposite direction to each other minimizing the steric hindrance between the methyl groups and increases contact between the O atom and the cation. The crystal packing shows that each Na^+^/K^+^ counter ion is coordinated to three adeninate anion molecules via the N1, N7 and N9 atoms of three purine rings (Figure 2a, Figure S18 for K‐Ade). Conversely, one adeninate anion is coordinated to three Na^+^ or K^+^ counter ions via the N1, N7 and N9 atoms. This in agreement with previous research showing that the coordination sites of the adeninate anion are predominantly N1, N9 and N7.^[24]^

The crystal structure of K‐Ade(18C6), Figure 2b, provides further information, showing the N3 and N9 atoms acting as a chelate in coordination with one K^+^ counter ion, having the ion positioned almost equally between the N3‐ and the N9‐atom. The K^+^ ion is simultaneously interacting with one 18C6 ether molecule and one adeninate anion. The adeninate anion molecules form zigzag ribbons along the a axis (Figure S19). The K^+^ and 18C6 coordination is slightly perched, with a *d* value of 0.924(8) Å (distance of K^+^ from the centroid of the 18C6). A herringbone packing morphology of the K‐Ade(18C6) units along the a axis is observed wherein a hydrogen‐bonded network exists in which the adeninate anion molecules are linked via a mixed WC and HG type hydrogen bonding. From the same set of crystals, a second morphology is obtained, SI Figure S20, in which there is positional disorder in the system. Note that no DMSO solvent is included in the crystals in the presence of crown ether.

From the above, the N3 and N9 atoms are the most likely preferred sites of coordination in solution as the positively charged counter ion will interact with two lone pairs on either nitrogen atom. The size dependency of the counter ion for the N3–N9 coordination will favor that of the K^+^ ion as it is larger in size and more suitable for the bridging between the two atoms. The Na^+^ ion can chelate or coordinate via either the N9 or the N3 site. No direct evidence for cation‐π interactions have been observed and the effect of the presence of the different counter ions on the ^13^C NMR chemical shifts is too large to be due to only electrostatic interactions between the π system and the counter ion. The difference between the ^13^C chemical shifts of K‐Ade and Na‐Ade range from 0.9–3.0 ppm for the imidazole ring whereas the difference between K^+^ and Na^+^ in a cation‐π interaction with benzene is predicted to be 0.13 ppm.^[43]^ It has been shown that compounds containing heteroatoms, such as imidazole, pyridine, aniline and imidazole have the cation interact with the heteroatom in preference to the aromatic system, with the cation‐π binding being significantly low for imidazole.^[44]^

### 2.3 Concentration and ^1^H NMR temperature coefficient of Na‐Ade, K‐Ade, Na‐Ade(15C5) and K‐Ade(18C6)

**Figure 3:**
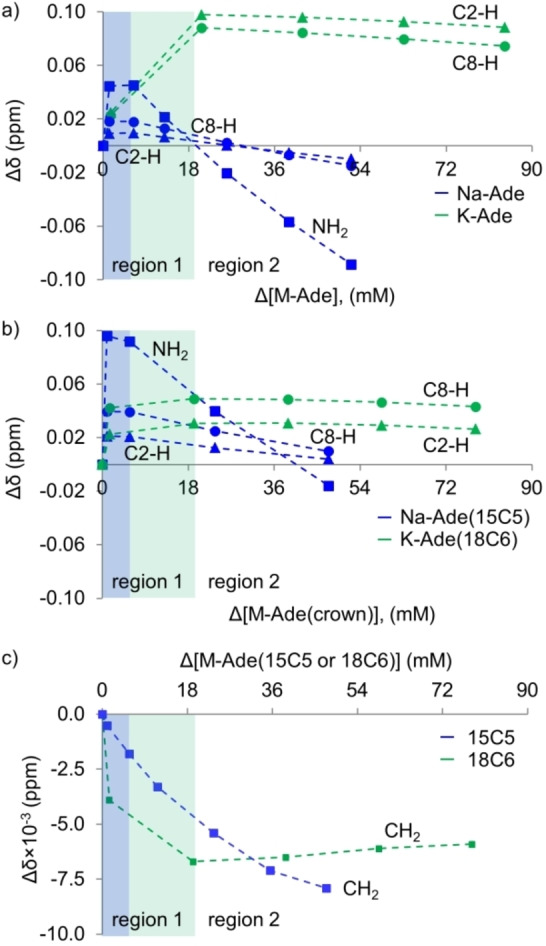
Change in chemical shift of purine protons with the change in concentration for a) Na‐Ade and K‐Ade, b) Na‐Ade(15C5) and K‐Ade(18C6), NH_2_ – squares, C8‐H – circles and C2‐H – triangles, and c) the CH_2_ protons of the 15C5 and 18C6 ether in the presence of Na‐Ade and K‐Ade, respectively. All in DMSO‐d_6_ at 320 K. Region 1 (highlighted in blue and green for Na‐Ade and K‐Ade, respectively) and region 2 indicates concentration dependent change in chemical shift.

Depending on the solvent used, hydrogen bonded (chloroform as a solvent) or stacked dimers (solvents such as water or DMSO) occur upon increasing concentrations of adenine, leading to the deshielding of the NH_2_ protons^[45]^ or shielding of the purine protons,^[46]^ respectively. The competing H⋅⋅⋅O interaction between the amino group of adenine with water has a larger interaction energy than H⋅⋅⋅N, promoting the formation of stacked dimers over hydrogen bonded dimers.[^7^, ^39^] DMSO should have a similar effect due to the partial negative oxygen available for hydrogen bonding. Concentration dependent ^1^H NMR studies were performed to investigate the ion‐dependent self‐aggregation of the ionic complexes (Figure S6a and b). To gain further knowledge of the influence of the cation and the effect of the potential complexation of the K‐Ade with 18C6, ^1^H NMR studies in the presence of 2.4 equivalents of 15C5 ether for Na‐Ade (Na‐Ade(15C5)) and 18C6 ether for K‐Ade (K‐Ade(18C6)), Figure S6c and d, were performed. A graphical representation of the shifts on a common axis is given in Figure S7. The change in ^1^H NMR chemical shifts with the change in concentration (Δδ=δ_0_‐δ_n_, where δ_0_ is the chemical shift of the reference point at the lowest concentration and δ_n_ of higher concentration) of the ionic complexes as well as the CH_2_ of the 15C5 and 18C6 are shown in Figure 3.

**Figure 4:**
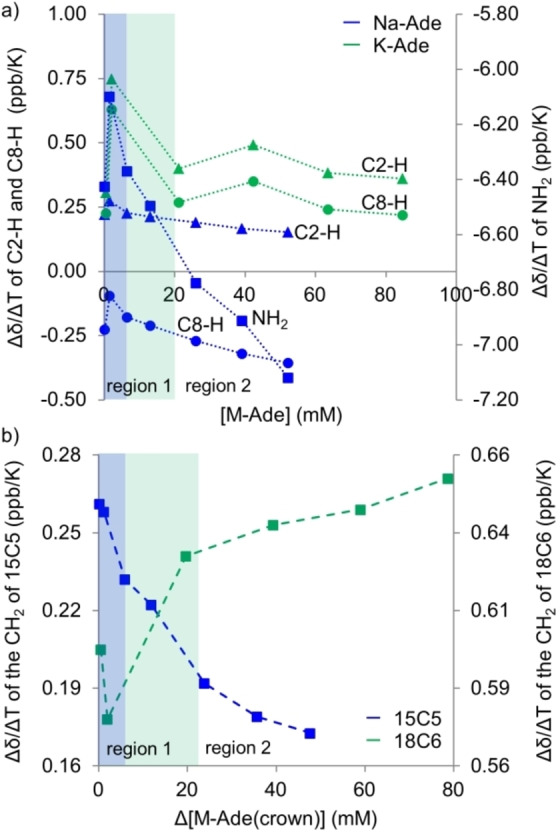
The change in temperature coefficients with concentration of ^1^H chemical shifts of a) Na‐Ade and K‐Ade and b) CH_2_ of 15‐crown‐5 ether and the 18‐crown‐6 ether in DMSO‐d_6_. NH_2_ (squares), C8‐H (circles) and C2‐H (triangles). The shading relates to the regions defined in Figure 3.

Temperature dependent studies were conducted over the range of concentrations used in order to investigate how increasing the temperature of the solution affects the interactions between molecules as the distance between them is increased. Refer to Figure S9 for the change in δ with temperature (300 K–380 K). The change in chemical shift of a proton with the change in temperature gives the temperature coefficient (Δδ/ΔT) which is used to monitor hydrogen bonding and π‐π stacking between molecules.[^45^, ^46^] Dissociation of hydrogen bonds leads to shielding of the involved protons and therefore a negative Δδ/ΔT value. Protons exposed to solvent molecules will experience a large negative temperature dependence of the chemical shift as the hydrogen bonds with the solvent are broken. The reduction in temperature coefficient with increasing concentration indicates hydrogen bond formation shielding protons from hydrogen bonding with solvent molecules, which in turn results in a decreased dependence of the proton chemical shift with temperature.^[45]^ For molecules in solution, as the dissociation energy of hydrogen bonds increases, the dependence of the chemical shift on temperature decreases as a larger amount of energy is needed to distort the bond.^[47]^ The effect of temperature on stacked dimers can also be studied using Δδ/ΔT.^[46]^ Dissociation of dimers involved in π‐π stacking results in the deshielding of purine protons and hence a positive temperature coefficient (Δδ/ΔT>0).^[46]^ The change in the temperature coefficient with concentration is shown for the purine protons of Na‐Ade and K‐Ade, Figure 4a, and for the CH_2_ protons of the 15C5 and 18C6 ether, Figure 4b. Refer to Figure S10 for the temperature coefficient of Na‐Ade(15C5) and K‐Ade(18C6).

### 2.4 Aggregation by π‐π Stacking and In‐Plane Dimerization

**Figure 5:**
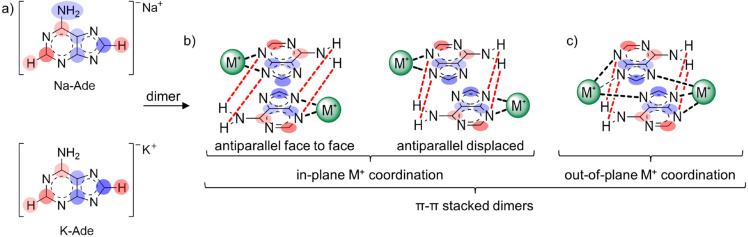
Cation induced change in atomic electron density in Na‐Ade and K‐Ade relative to adenine, based on the NMR chemical shifts in Table 2. Two shades of blue indicate shifts towards more positive, red towards more negative charge. b) Suggested structures of π‐π stacked, c) proposed shift of the M^+^ ion from in‐plane coordination to an out‐of‐plane coordination in the π‐π stacked dimer of M‐Ade. Hydrogen bonding indicated by red lines.

Two concentration dependent regions with opposite change in chemical shift are observed in Figure 3a, indicated as region 1 and region 2. Region 1 showed shielding of both purine protons, which indicates π‐π stacking of the ionic complexes and is attributed to the ring current effect experienced by the neighboring molecule in π‐π stacked dimers. The observed changes in region 1 are not explained by ion‐pairing which would have caused deshielding of purine protons. Highlighting the gain and loss of electron density in the ^13^C NMR chemical shifts of Na‐ and K‐Ade relative to adenine, Figure 5a, it is clear that the purine ring is partially positive in the imidazole ring and negative in the pyrimidine ring. In the presence of competitive hydrogen bonding with DMSO solvent molecules, the dimerization between two M‐Ade molecules would be driven by π‐π stacking due to stabilizing electrostatic interactions between the oppositely charged sections of the purine ring, having the 5 membered ring of one molecule stack with the 6 membered ring of the neighboring molecule. This can occur via either antiparallel face to face or antiparallel displaced stacking, Figure 5b. Anti‐parallel stacking has been suggested for deprotonated purines.^[48]^ These stacked dimers can be further enhanced by hydrogen bonding of the NH_2_ group with the N3 and N9 atom on the neighboring ring and additional stabilization can occur if the counter ion moves from in‐plane to out‐of‐plane coordination (Figure 5c) allowing for a bridging interaction with both adeninate anion molecules, coordinating to the N3, N9 and N7 atoms. The larger sized K^+^ counter ion could bridge between two neighboring molecules more readily than the smaller, more tightly bound Na^+^ counter ion. In aqueous media, the self‐association of nucleotide bases is driven by π‐π stacking in which the association constants for nucleobases involved are in the order of purine‐purine>purine‐pyrimidine>pyrimidine‐pyrimidine.^[49]^ Therefore, at low concentrations, with competitive hydrogen bonding to DMSO solvent molecules, π‐π stacking of the adenine salts is highly likely to be the dominant form of aggregation.

The absence of the NH_2_ peak at all concentrations in the ^1^H NMR of K‐Ade indicates that the interaction of K^+^ with the adeninate anion is different from that of Na^+^. Thus, an alternative protonation state or proton exchange regime of the NH_2_ group of K‐Ade, either with the hydrogen bonded DMSO or neighboring adeninate molecules, could provide an explanation as to why the protons are not observed in the ^1^H NMR spectra. The nuclear electric quadrupole moment of K^+^ is smaller than that of Na^+^, so its coupling with the hydrogen atoms is unlikely the reason for an additional relaxation. If the π‐π stacked dimer does indeed occur, this phenomenon could be supported as the K^+^ counter ion would be closer in the vicinity of the hydrogen atoms or shared or in a dynamic equilibrium between two adeninate anions.

The addition of 15C5 ether to Na‐Ade did not result in a large difference in ^1^H NMR chemical shifts concluding that it did not completely interact with the Na^+^ from Na‐Ade. The ability of the solvent to solvate the cation will influence the association constant for the M^+^‐crown formation.^[50]^ The Na^+^(15C5) complex is a perched complex allowing for the Na^+^ to interact with solvent molecules.^[51]^ Therefore, in the presence of DMSO solvent molecules and the highly competing adeninate anion, complexation between Na^+^ and 15C5 ether will be significantly lowered.

**Scheme 1:**
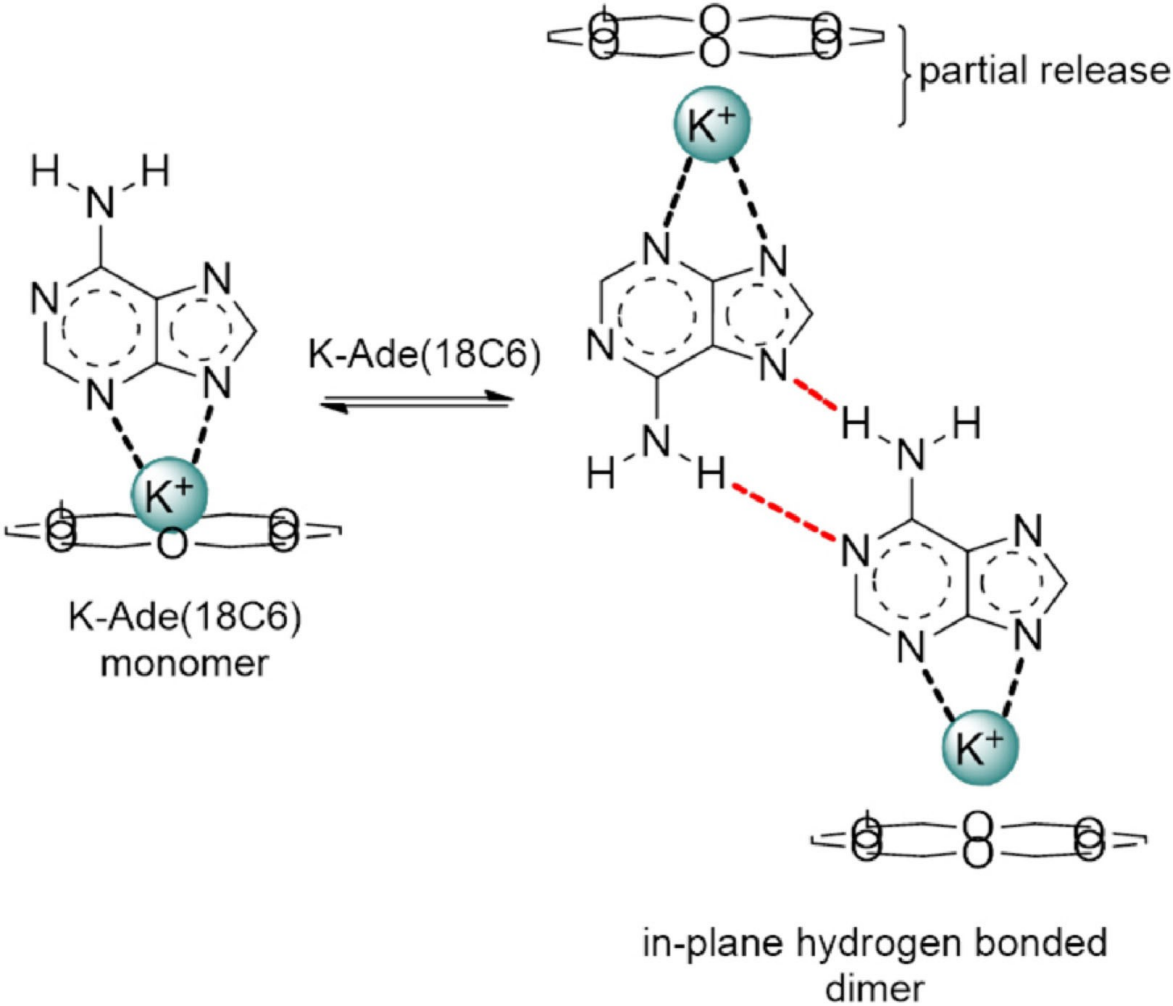
Hydrogen bonded aggregates of K‐Ade(18C6). Hydrogen bonding shown by red dashed line.

The addition of 18C6 to K‐Ade resulted in a reduced concentration dependence in region 1, Figure 3b, and at the very dilute concentrations of region 1, the ^1^H NMR peak of the NH_2_ protons became visible (Figure S4 insert). The 18C6 complexed with the K^+^ forming a new complex whereby the K^+^ ion is simultaneously interacting with the 18C6 and the adeninate anion. The crystal structure of K‐Ade(18C6) reveals the feasibility of such an interaction and is supported by the lack of evidence for removal of the K^+^ in the ^1^H and ^13^C NMR spectra (the UV data on the other hand, was obtained in dilute solutions with >100‐fold excess of the crown to allow for removal of the counter ion). The K^+^(18C6) complex is destabilized by the presence of the DMSO solvent molecules or any competitive complexing molecule, such as the adeninate anion, and hence the K^+^ will not complex to the 18C6 ether alone. The formation of an adeninate‐K^+^‐18C6 complex is likely to disfavor aggregation via π‐π stacking. Thus, in region 1 of Figure 3b, monomers of K‐Ade(18C6) complex are likely the predominant species. This would potentially allow for the NH_2_ peak of K‐Ade(18C6) to become visible since K^+^ would not be in the vicinity of the NH_2_ group such as in the proposed π‐π stacked dimers. The increased shielding of the purine protons with concentration in region 1 (Figure 3b) can be linked to the gain of electron density in the purine ring as the 18C6 ether complexes with the K^+^ of K‐Ade, reducing the level of interaction between the adeninate anion and K^+^. In‐plane mixed WC‐HG hydrogen bonded dimers such as those observed in the XRD crystal structure of K‐Ade(18C6) might occur in region 2 (Figure 2b). Evidence of abstraction of the K^+^ from the 18C6 in Figure 3c region 2, CH_2_ protons become shielded as electron density is regained, suggests influence of the aggregate formation on the interaction between K^+^ and 18C6 ether, resulting in partial release, Scheme 1. Upon increasing the temperature and shifting the equilibrium towards the monomer form, the 18C6 would engage in tighter binding once again as suggested by the increased temperature coefficient in Figure 4b showing increased interaction between the K^+^ ions and the 18C6. The absence of the NH_2_ peak at higher concentrations could be a result of imine formation involving significant dynamics of chemical shifts. From the crystal structure of K‐Ade(18C6), the hydrogen bond lengths between the NH⋅⋅⋅N7 and NH⋅⋅⋅N1 differ between the linked molecules, having the latter differ quite significantly, 2.262 vs 2.160 Å, which could arise from imine formation.

### 2.5 Higher Order Aggregates

**Scheme 2:**
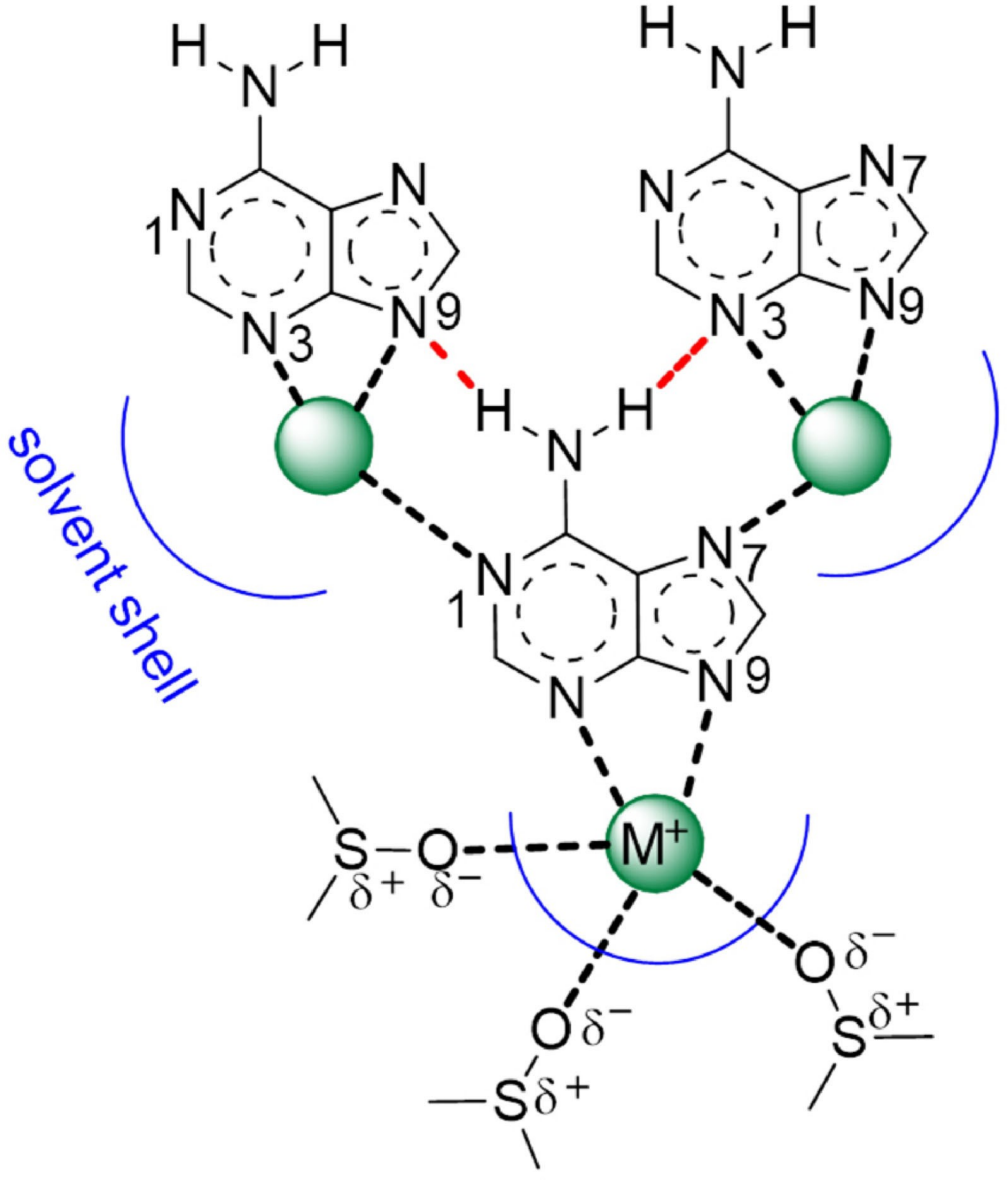
M‐Ade aggregate formation via the N1 and/or the N7.

Apart from providing insight into the site of coordination between the counter ion and the adeninate anion, the crystal structures provide an interesting comparison and perhaps even some guidance for the aggregate structures of Na‐ and K‐Ade in solution. The Na‐ and K‐Ade complexes in the crystal structures do not show in‐plane adeninate‐adeninate anion mixed WC‐HG hydrogen bonded aggregates as observed in K‐Ade(18C6), nor are π‐π stacked aggregates evident. Instead, the Na‐ and K‐Ade complexes are linked via the counter ions. Hydrogen bonds occur between the NH_2_ of one molecule with an N3 and N7 of two neighboring molecules. This could suggest that higher order aggregate formation in region 2 of Figure 3a are governed by the complexation of M‐Ade molecules via the counter ions present, Scheme 2, through the coordination of N1 or N7 to the counter ion of the neighboring M‐Ade molecule. Aggregation of this type could result in the migration of the counter ion between the N3 or N9 position allowing the N9 or N3, respectively, to be available for hydrogen bonding. Dissociation of these aggregates with increased temperature would result in shielding of the NH_2_ protons involved in the hydrogen bond with the N3/N9 of the neighboring molecule, and i. e. shielding of the C8‐H and deshielding of the C2‐H as the counter ion moves back from the N9 into the N3N9 coordination site, as observed with the temperature coefficient in Figure 4a. It has been suggested that the C8‐H could be involved in hydrogen bonding with DMSO,^[52]^ therefore, this effect has not been ruled out as additional influence on the chemical shift.

The concentration dependent change in the temperature coefficient in Figure 4a for K‐Ade showed both purine protons undergo deshielding in region 2 and decreases with increasing concentration. This effect is ascribed to the temperature dependent solvent electric field effect as no explanation can be provided considering the disruption of hydrogen bonds. The cation has a peripheral solvation shell of DMSO solvent molecules which orient their dipoles towards the cation, Scheme 2. The positive charge on the cation would be partially reduced as an inflow of electron density from the partially negative O atom of DMSO will occur, weakening the ionic bond with the adeninate anion. An increase in temperature results in the disorder of these arranged DMSO molecules,^[53]^ strengthening the ionic bond between K^+^ and the adeninate anion, leading to deshielding of both purine protons. If the higher order aggregates in region 2 shield the cations from the solvent, this effect would reduce with increased concentration, which is what is observed with the reduced temperature coefficient of K‐Ade. The proposed aggregate formation is one whereby one K‐Ade coordinates via the N7 to the K^+^ of the neighboring K‐Ade molecule which then has another neighboring molecule coordinate via the N1. This type of aggregation will begin to shield the counter ion from the solvent, reducing the solvent electric field effect.

The above proposed aggregate formations do not rule out the π‐π stacking believed to occur at the lower concentrations of region 1 as the complexation of the ionic complexes in higher order aggregates in region 2 can disrupt the aggregation in region 1. Adenine has been reported to form trimers and higher order aggregates upon self‐association.^[8]^ Dimer formation has been observed for adenine, adenosine and adenosine monophosphate at concentrations lower than 5 mM, followed by higher order aggregation at concentrations >5 mM ascribed to the polymerization of dimers.^[54]^ This provides confidence that dimer formation can occur at low concentrations (region 1), followed by the formation of higher order aggregates with increasing concentration (region 2).

### 2.6 Thermodynamics of Self‐Aggregation

Two processes are possible for a single adeninate anion, the first is the ion‐pairing between the adeninate anion and the counter ion Na^+^/K^+^ and the second is the self‐aggregation of the ion pairs. It is assumed here that i) the self‐complexation between free adeninate anions is negligible and ii) a significant amount of ion‐pairing already exists. The concentration dependence of the chemical shifts observed in Figure 3 for Na‐/K‐Ade, Na‐Ade(15C5) and K‐Ade(18C6), is ascribed to self‐aggregation and can occur via monomeric metal‐adeninate (M‐Ade) addition, i. e. monomer→dimer→trimer etc. The isodesmic and trimer models were used to fit the normalized ^1^H NMR data of region 2 (refer to Table S1 in SI for K vlaues). In addition, the thermodynamic data for the 1 : 1 complexation between 15C5 ether with the Na^+^ were obtained (Table S2). This was not possible for the complexation between 18C6 ether with K^+^.

The van't Hoff plot for the self–aggregation of Na‐Ade, Na‐Ade(15C5) and K‐Ade and for the 1 : 1 complexation between 15C5 ether and Na^+^ is shown in Figure S15. The thermodynamic data are given in Table 3.

**Table 3:** Aggregation constants and thermodynamic parameters for the self‐aggregation of Na‐Ade^a^, Na‐Ade(15C5)^[a]^ and K‐Ade^[b]^ and the association constant for the 1 : 1 complexation^[c]^ between C15C and Na^+^ in DMSO‐d_6_ as solvent at 305 K.

Complex	K_agg_ [M^−1^]	ΔH^[d]^	ΔS^[e]^	ΔG^[d]^
Na‐Ade	2.4±0.3	−4.7±0.2	−8.0±0.5	−2.2
Na‐Ade(15C5)	3.0±0.5	−5.2±0.5	−8.3±1.6	−2.7
	K (M^−2^)			
K‐Ade	0.59±0.03	−4.5±0.3	−19±1	+1.4
	K_1_ (M^−1^)			
Na‐Ade(15C5)	28±3	−23±2	−50±5	−8.4

[a] Isodesmic model, [b] Trimer model, [c] 1 : 1 Guest‐host model, [d] kJ mol^−1^, [e] J mol^−1^ K^−1^.

The equilibrium constants of aggregation are small and dominated by enthalpic interactions in the order of −5 kJ mol^−1^, which compares with a weak water‐amide N⋅⋅⋅H hydrogen bond and is smaller than that of the self‐aggregation of 9‐ethyladenine[^55^, ^56^] which has an enthalpy of formation of −16.7 kJ mol^−1^ associated with more than one hydrogen bond. Cyclic dimer formation (two hydrogen bonds involved in the dimer) is largely preferred over linear dimers (one hydrogen bond).^[5]^ Considering the above proposed ion‐assisted aggregate formation, only one hydrogen bond is involved between two M‐Ade molecules and it can therefore be expected that the enthalpy of formation will be about half. The gain in enthalpy is in part compensated by negative entropies of association of about −8 J mol^−1^ K^−1^, which is much less than the value of −145 J mol^−1^ K^−1^ that should typically be expected for the loss of translational entropy of a Na‐Ade unit due to conformational order.^[57]^ Much of it must be compensated by a large gain in entropy of liberated solvent molecules during aggregation of Na‐Ade and the gain in vibrational entropy^[58]^ from the formed weak hydrogen bond which would result in loose NH vibration. In addition, if the π‐π stacked dimer, which would have more order, is dissociated upon larger aggregate formation, this would also increase the entropy. The equilibrium of the K‐Ade aggregate is slightly disfavored as seen by the slight positive ΔG.

The change in enthalpy (ΔH=−23 kJ mol^−1^) for the 1 : 1 complexation between Na^+^ and 15C5 ether is slightly larger than that previously reported in DMF^[59]^ and DMSO^[60]^ (ΔH=−20 and −15 kJ mol^−1^, respectively) and is attributed to the use of anhydrous DMSO‐d_6_. The presence of water significantly reduces the binding of Na^+^ to 15C5 ether.^[60]^

## 3 Conclusions

Disfavored tautomeric and anionic forms of nucleobases which have gained attention recently surround their involvement in mutations, replication, and translational errors as well as nucleic acid catalysis and RNA‐ligand recognition. However, there is a lack of information surrounding the anionic forms of nucleobases such as the adeninate anion. In this paper, the ion‐pairing and self‐aggregation ability of the adeninate anion in DMSO are investigated using NMR and UV spectroscopy and crystallographic methods. An understanding of the variability of chemical shifts is needed in context of ongoing kinetic experiments by NMR.

The large ion‐dependent difference in the UV absorbance λ_max_ from 272 nm for K‐Ade to 269 nm for Na‐Ade shows that ion‐pairs exist between the adeninate anion and Na^+^ and K^+^ counter ions in DMSO at very dilute concentrations. The crystal structures obtained from the ionic complexes have the ion coordinate at the N1, N9 and N7 atoms of the purine ring. It is proposed that a chelating N3–N9 coordination is highly likely to exist in solution. The counter ions are not successfully removed by the addition of 15‐crown‐5 and 18‐crown‐6 ether from the Na‐ and K‐Ade as no gain of electron density in the purine system was observed in the ^1^H and ^13^C NMR study. It is proposed that the addition of 18‐crown‐6 ether results in complex formation with the K‐Ade, as indicated also by the crystal structure of K‐Ade(18C6).

In the concentration dependent ^1^H NMR studies, two regions with opposite chemical shift are observed which indicate two aggregation steps. The first aggregation is ascribed to dimer formation via π‐π stacking as suggested by the shielding of the purine protons by the ring current of the neighboring stacked molecule. At higher concentrations, the dimer formation is followed by higher order aggregate formation of the Na‐ and K‐Ade. The counter ion plays a role in the self‐aggregation allowing for coordination to the counter ion of the neighboring Na‐/K‐Ade ion pairs. The size of the counter ion influences how the aggregates are arranged or propagated though space, revealing a clear difference between the Na‐Ade and K‐Ade forms. The assembly of the Na‐ and K‐Ade complexes in the crystal structures does not show WC or HG type arrangement of hydrogen bonded aggregates. The possibility of weak hydrogen bond formation involving the NH_2_ is indicated by the small enthalpy of formation of about −5 kJ mol^−1^. The small decrease in entropy of about −8 J mol^−1^ K^−1^ with aggregate formation indicates that there is a gain in entropy due to either the liberation of solvent molecules, rearrangement of dimer aggregates and/or there is a gain in vibrational entropy from the formation of weak hydrogen bonds.

Theoretical studies have been conducted to investigate the proposed sites of coordination between the counter ions and the adeninate molecule (publication in progress). Current studies are underway to confirm the modes of aggregation, with this research providing a good starting point. Although these studies have been conducted in a non‐aqueous environment, they provide insight into how the existence of the potential deprotonated adenine base can interact in the alkali metal ions present, having the dielectric constant of DMSO fall in between that within the DNA double helix and inside the cellular environment.^[37]^

The counter ion present plays an essential role in directing aggregate formation, adding new insight into how the adeninate anion interacts with neighboring molecules, and hence provides information regarding the reactivity of the systems as well as insight for the association of adenine with alkali metal counter ions which can provide stability for these forms.

## Conflict of interest

The authors declare no conflict of interest.

## Supporting information

As a service to our authors and readers, this journal provides supporting information supplied by the authors. Such materials are peer reviewed and may be re‐organized for online delivery, but are not copy‐edited or typeset. Technical support issues arising from supporting information (other than missing files) should be addressed to the authors.

Supporting Information
