# Supplementary material for: Towards a Molecular Understanding of Cation‐Anion Interactions and Self‐aggregation of Adeninate Salts in DMSO by NMR and UV Spectroscopy and Crystallography
Source: Chemphyschem. 2021 Aug 8;22(19):2025–33. doi: 10.1002/cphc.202100098 (PMC8518609; doi:10.1002/cphc.202100098)
Supplement: Supplementary file 1 — Supporting Information [file CPHC-22-2025-s001.pdf]

ChemPhysChem

Supporting Information

Towards a Molecular Understanding of Cation-Anion Interactions and Self-aggregation of Adeninate Salts in DMSO by NMR and UV Spectroscopy and Crystallography

Dominique M. S. Buyens, Lynne A. Pilcher, and Emil Roduner*

Author Contributions

E.R. Conceptualization:Supporting; Supervision:Lead; Writing – review & editing:Equal
D.B. Conceptualization:Lead; Data curation:Lead; Formal analysis:Lead; Methodology:Lead; Project administration:Equal; Validation:Lead; Visualization:Lead; Writing – original draft:Lead
L.P. Conceptualization:Equal; Funding acquisition:Lead; Project administration:Lead; Supervision:Supporting; Writing – review & editing:Equal

Experimental details

General: The ^{1}H NMR spectra were recorded at 300 MHz with a Bruker 300 AVANCE Ultrashield Plus. Heteronuclear Multiple Bond Correlation (HMBC) spectra were recorded at 400.13 MHz with a Bruker 400 AVANCE Ultrashield Plus. The ^{13}C NMR spectra were recorded at 101 MHz using the Bruker 400 AVANCE Ultrashield Plus. The ^{13}C NMR spectra were calibrated using the DMSO-d_6 solvent peak at 39.5 ppm. Adenine, sodium and potassium hydride (purified from 60% in dispersion mineral oil by hexane), and DMSO-d_6 were purchased from Sigma-Aldrich. Stock solutions and dilutions of Na-Ade, K-Ade, Na-Ade(15C5) and K-Ade(18C6) were prepared within the glovebox using the anhydrous DMSO-d_6. Details for stock concentrations and dilutions are given in the SI. The UV absorbance spectra were measured using Varian Cary 300 UV–Vis spectrophotometer scanning from 200 to 800 nm. Samples were run in a 1 mm quartz cuvette for DMSO samples with DMSO as a blank sample, and a 4 mm quartz cuvette for anhydrous DMSO samples (prepared and sealed in a glove box) with anhydrous DMSO as the blank sample. Single crystal X-ray diffraction analysis of compounds Na-Ade, K-Ade and K-Ade(18C6) were performed on a Rigaku XtaLAB Synergy R diffractometer and a Bruker D8 Venture kappa geometry diffractometer, refer to the SI for more information.

Materials and Methods

Experimental details for ^{1}H NMR studies and crystallography

The anhydrous DMSO-d_6 was prepared as follows: Powdered 4 Å molecular sieves were heated at 200 °C under vacuum for 4 days and placed inside a glovebox. The sieves were added to DMSO-d_6 and left for 24 hours. The solvent was analyzed via ^{1}H NMR to confirm the absence of water. The molecular sieves were removed by passing the solvent through a 25 mm hydrophilic polyamide syringe filter.

Purification of 18-Crown-6 via recrystallization: A mixture of 18-crown-6 and acetonitrile was heated until 18-crown-6 fully dissolved. Clear crystals formed when the heated mixture was allowed to cool to ambient temperature and left for 1 hr and then placed in the freezer for an additional hour. The crystals were filtered, washed with cold acetonitrile and vacuum dried over 24 hrs at 40 °C.[1]

Preparation of adenine-salt stocks

The sodium and potassium hydride are used as bases to avoid introduction of water into the reactions.

Preparation of Na-Ade stock: NaH (0.0688 mmol, 1.20 eq.) was added to a heterogeneous mixture of adenine (0.0574 mmol, 1 eq.) and anhydrous DMSO-d_6 (1100 μl). 7 concentrations were prepared, A (52.2), B (39.1), C (26.1), D (13.0), E (6.52), F (1.44), G (0.130).

Preparation of K-Ade stock: KH (0.121 mmol, 1.30 eq.) was added to a heterogeneous mixture of adenine (0.0932 mmol, 1 eq.) and anhydrous DMSO-d_6 (1100 μl). 6 concentrations were prepared, A (84.8), B (63.6), C (42.4), D (21.2), E (2.12), F (0.207).

Preparation of K-Ade(18C6) stock: 18-crown-6 (0.320 mmol, 2.40 eq.) was added to a solution of adenine (0.134 mmol, 1 eq.) and KH (0.160 mmol, 1.19 eq.) in anhydrous DMSO-d_6 (1632 μl). A precipitate formed which was filtered off using 25 mm hydrophilic polyamide syringe filter. From this, 6 concentrations were prepared, A (78.7), B (59.0), C (39.3), D (19.7), E (1.97), F (0.489).

Preparation of Na-Ade(15C5): 15-crown-5 (0.126 mmol, 2.40 eq.) was added to a solution of adenine (0.0523 mmol, 1 eq.) and NaH (0.0629 mmol, 1.20 eq.) in anhydrous DMSO-d_6 (1075 μl). From this, 7 concentrations were prepared, A (47.6), B (35.7), C (23.8), D (11.9), E (5.95), F (1.19), G (0.238).

Experimental details for UV spectroscopy studies

An estimation of the content of water in DMSO from the bottle that has been open was calculated using an internal standard of toluic acid in non-anhydrous DMSO-d_6. Both bottles have been kept in a desiccator with dried silica gel and under N_2 gas. The content of water in 0.5 ml of DMSO-d_6 was found to be 5.2%.

Preparation of Na-Ade: Adenine (0.0500 mmol, 1 eq.) and NaH (0.0603 mmol, 1.20 eq.) were added to 1500 μl DMSO. From this, 12 concentrations were made: A (1.00), B (0.900), C (0.800), D (0.560), E (0.392), F (0.274), G (0.192), H (0.135), I (0.0941), J (0.0659), K (0.0461), L (0.0323) mM. These measurements were made using a 1 mm quartz cuvette. Anhydrous samples were prepared in a similar fashion in a glovebox using dried DMSO as the solvent. These sample measurements were made using a 4 mm quartz cuvette which was sealed in the glovebox. The Na-Ade(15C5) sample was prepared by addition of 126 eq of 15C5 ether to a final concentration of 0.33 mM of Na-Ade in DMSO. The measurement was done using a 1 mm quartz cuvette.

Preparation of K-Ade: Adenine (0.0500 mmol, 1 eq.) and KH (0.0605, 1.2 eq.) were added to 1000 μl DMSO. From this, 12 concentrations were made: A (1.00), B (0.900), C (0.800), D (0.560), E (0.392), F (0.274), G (0.192), H (0.135), I (0.0941), J (0.0659), K (0.0461), L (0.0323) mM. These measurements were made using a 1 mm quartz cuvette. Anhydrous samples were prepared in a similar fashion in a glovebox using dried DMSO as the solvent. These sample measurements were made using a 4 mm quartz cuvette which was sealed in the glovebox. K-Ade(18C6) sample was prepared by addition of 117 eq of 18C6 ether to a final concentration of 0.33 mM of K-Ade in DMSO. The measurement was done using a 1 mm quartz cuvette.

X-ray crystallography of Na-Ade, K-Ade and K-Ade(18C6)

Single crystal diffraction experiments of compounds Na-Ade, K-Ade and K-Ade(18C6) (morphology 2 in the SI) were performed on a Rigaku XtaLAB Synergy R diffractometer, with a rotating-anode X-ray source and a HyPix CCD detector. Data reduction and absorption were carried out using the CrysAlisPro (version 1.171.40.23a) software package.[2] Single crystals of K-Ade(18C6) (morphology 1 in the main text) was analyzed using Quazar multi-layer optics monochromated Cu Kα radiation (k = 0. 1.54184 Å) on a Bruker D8 Venture kappa geometry diffractometer with duo Iμs sources, a Photon 100 CMOS detector and APEX III control software.[3] Data reduction was performed using SAINT+,[3] and the intensities were corrected for absorption using SADABS-2016.[3] All X-ray diffraction measurements were performed at 150(1) K, using an Oxford Cryogenics Cryostat. All structures were solved by direct methods

with SHELXT-2018[4] and refined using SHELXL-2018 packages[5]. All H atoms were placed in geometrically idealised positions and constrained to ride on their parent atoms. For data collection and refinement parameters, see the Table S3. The X-ray crystallographic coordinates for the structures Na-Ade and K-Ade(18C6) have been deposited at the Cambridge Crystallographic Data Centre (CCDC), with deposition numbers CCDC 2055270-2055272. The X-ray crystallographic coordinates for the structure K-Ade were not submitted as the data was poor.

HMBC NMR for characterization of proton peaks of adenine-Na, adenine-K and adenine-K 18-crown-6

Assignment of the purine protons of Na-Ade, K-Ade and K-Ade(18C6) were achieved using HMBC, Figures S1-S3.

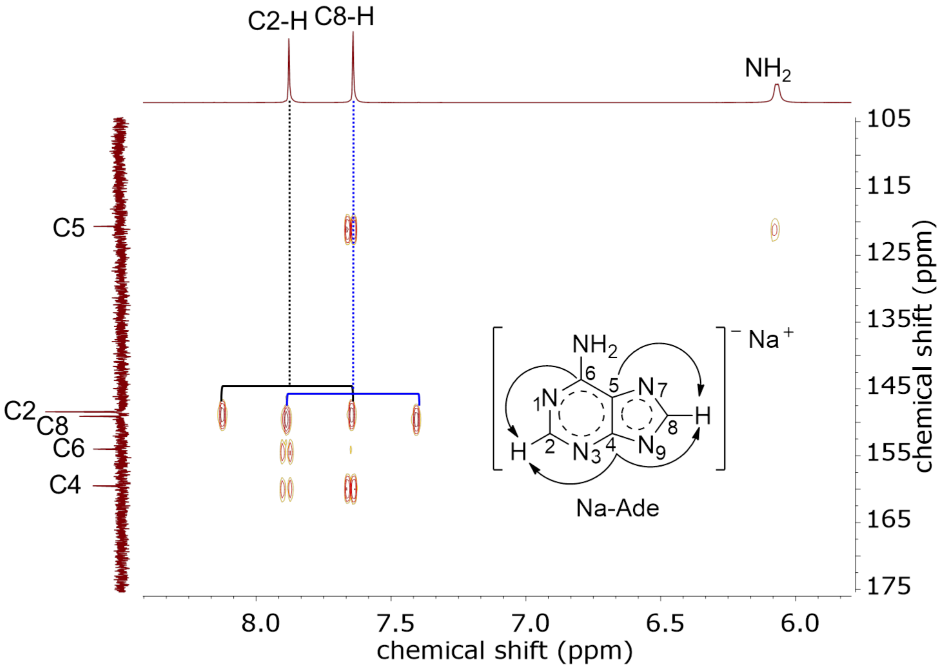

Figure S1: HMBC of adenine-sodium salt in anhydrous DMSO-d_6. Arrows indicate the observed carbon correlation with protons 2 or more bonds away. Black and blue dotted line show hovering correlation with the directly bonded proton.

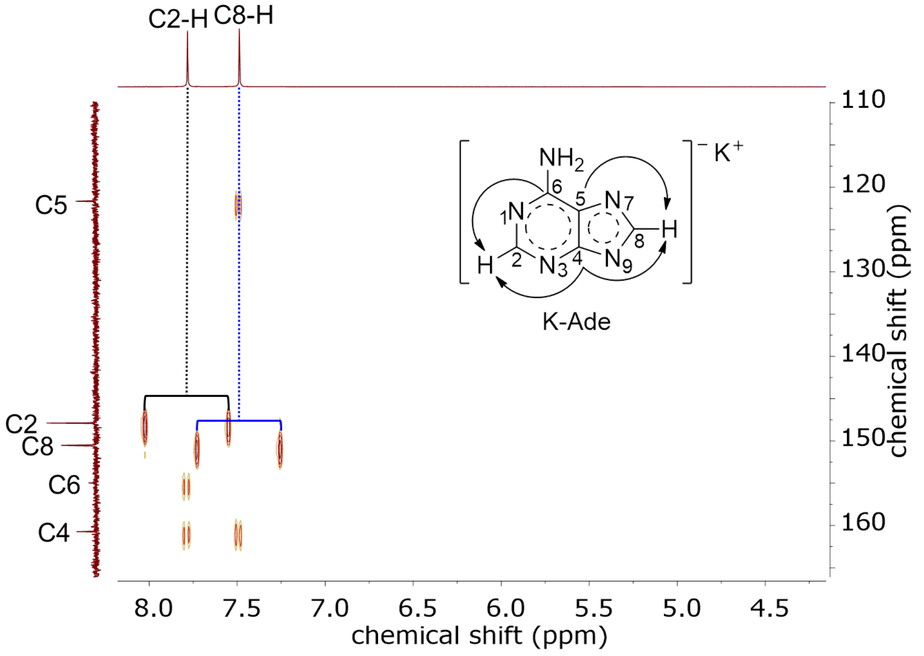

Figure S2: HMBC of adenine-potassium salt in anhydrous DMSO-d_6. Arrows indicate the observed carbon correlation with protons 2 or more bonds away. Black and blue dotted line show hovering correlation with the directly bonded proton.

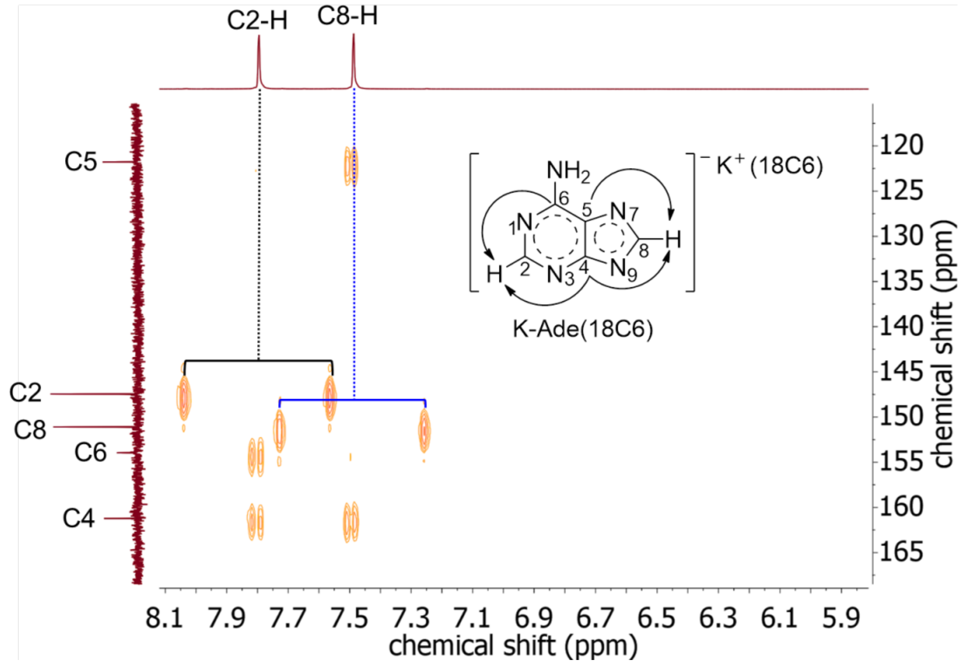

Figure S3: HMBC of adenine-potassium salt with 18-crown-6 in anhydrous DMSO-d_6. Arrows indicate the observed carbon correlation with protons 2 or more bonds away. Black and blue dotted line show hovering correlation with the directly bonded proton.

The superimposed ^{1}H NMR spectra of Na-Ade, K-Ade and K-Ade(18C6) in anhydrous DMSO-d_6 at the lowest concentrations used in the NMR study are shown in Figure S4.

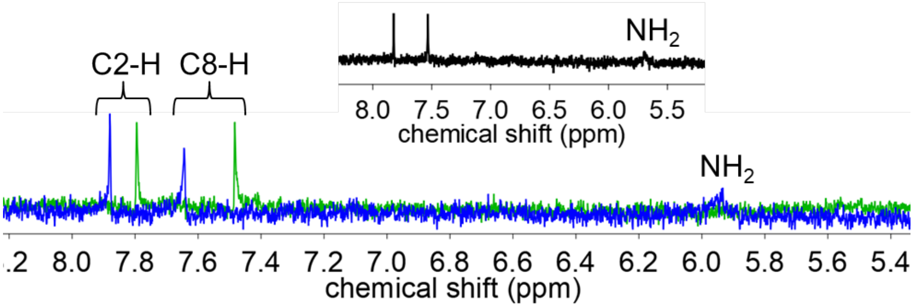

Figure S4: Superimposed ^{1}H NMR spectra of Na-Ade (blue, 1.44 mM), K-Ade (green, 2.11 mM) and K-Ade(18C6) (black, 0.489 mM) in anhydrous DMSO-d_6

The most likely site for the metal ion coordination is the N3-N9 and N9 for K^+ and the N3-N9, N3 and N9 for the smaller Na^+ ion, Figure S5. The nitrogen(s) directly involved in the ionic bond will donate electron density to the counter ion, and therefore "pull" electron density from the surrounding nitrogen and carbon atoms. This effect will affect the electron density of the directly associated ring and hence decrease the effect of the ring current resulting in a lessened deshielding effect (solid arrows). The neighbouring ring will experience this affect to a smaller extent (dashed arrows).

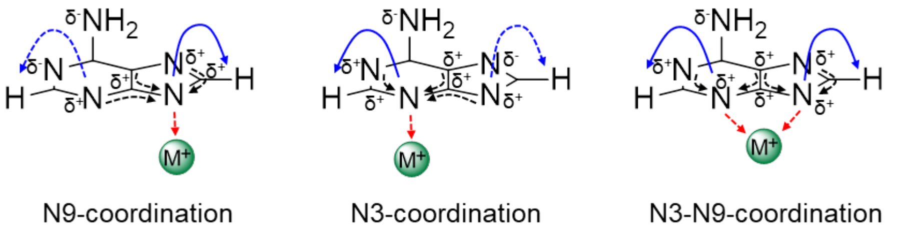

Figure S5: Metal ion coordinating to the N9, N3 and N3-N9

In turn, the shielding of the carbon atoms in the ^{13}C NMR will also be influenced by the coordination of the metal ion to the nitrogen atoms. The carbon atoms directly neighbouring the involved nitrogen will be more deshielded relative to adenine. The K-Ade has the C4, C5 and C8 more deshielded then the Na-Ade. This is attributed to the K^+ coordinating to the N3-N9 or N9 position. For Na-Ade, the Na^+ could coordinate either at the N3, N3-N9 or N9 atom and therefore the ^{13}C NMR will be an average between the three. As the coordination to the N3 will allow for the N7 atom to donate less electron density, the C8 and C5 will be more shielded on average.

The ^{1}H and ^{13}C NMR chemical shift positions are also influenced by the aggregation and therefore these relative shifts discussed above and the proposed reasoning is only a part of the explanation.

A set of concentrations were used to investigate the aggregation behaviour of Na-, K-Ade. Na-Ade(15C5) and K-Ade(18C6) in anhydrous DMSO-d_6. The concentration dependent ^{1}H NMR spectra of Na-, K-Ade, Na-Ade(15C5) and K-Ade(18C6) are shown in Figure S6a-d.

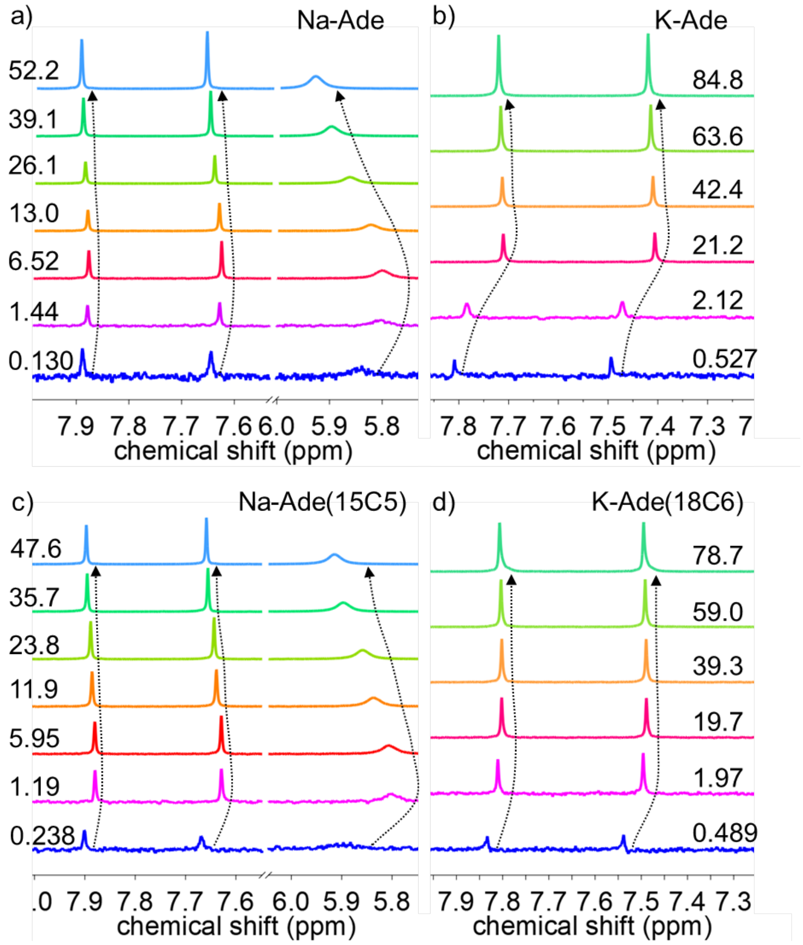

Figure S6: ^{1}H NMR spectra of a) Na-Ade, b) K-Ade c) Na-Ade(15C5) and d) K-Ade(18C6) at the indicated concentrations (mM) in anhydrous DMSO-d_6 at 320 K. The vertical lines are drawn as a guide to the eye.

A graphical representation of the above shifts on a common axis is given in Figure S7 and reveals that for Na-Ade and Na-Ade(15C5), the proton chemical shifts are roughly the same whereas for K-Ade and K-Ade(18C6) they are different. Hence, the nature of the adeninate anion of K-Ade changes much more in the presence of the crown ether than it does for Na-Ade.

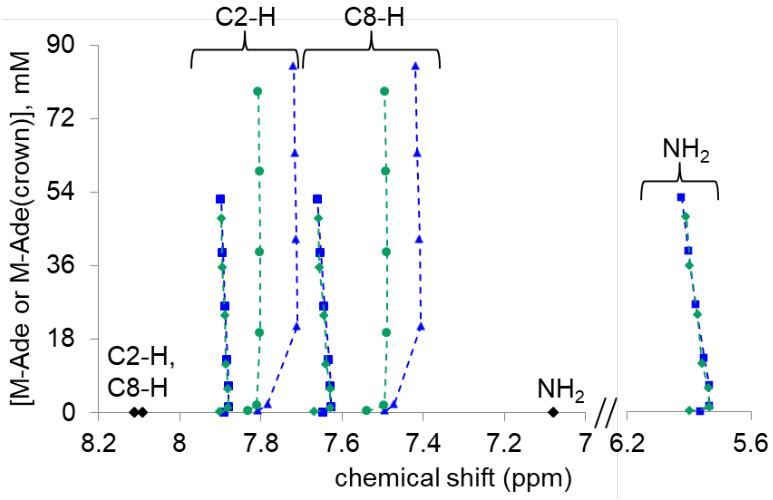

Figure S7: Graphical representation of the concentration dependent ^{1}H NMR chemical shift for the indicated protons of Na-Ade (blue squares), Na-Ade(15C5) (green diamonds), K-Ade (blue triangles) and K-Ade(18C6) (green circles). Chemical shifts of adenine purine protons are shown in black for comparison purpose.

The concentration dependent ^{13}C NMR spectra for the reported region 2 concentration range in Figure 3, main text, is shown in Figure S8a and b.

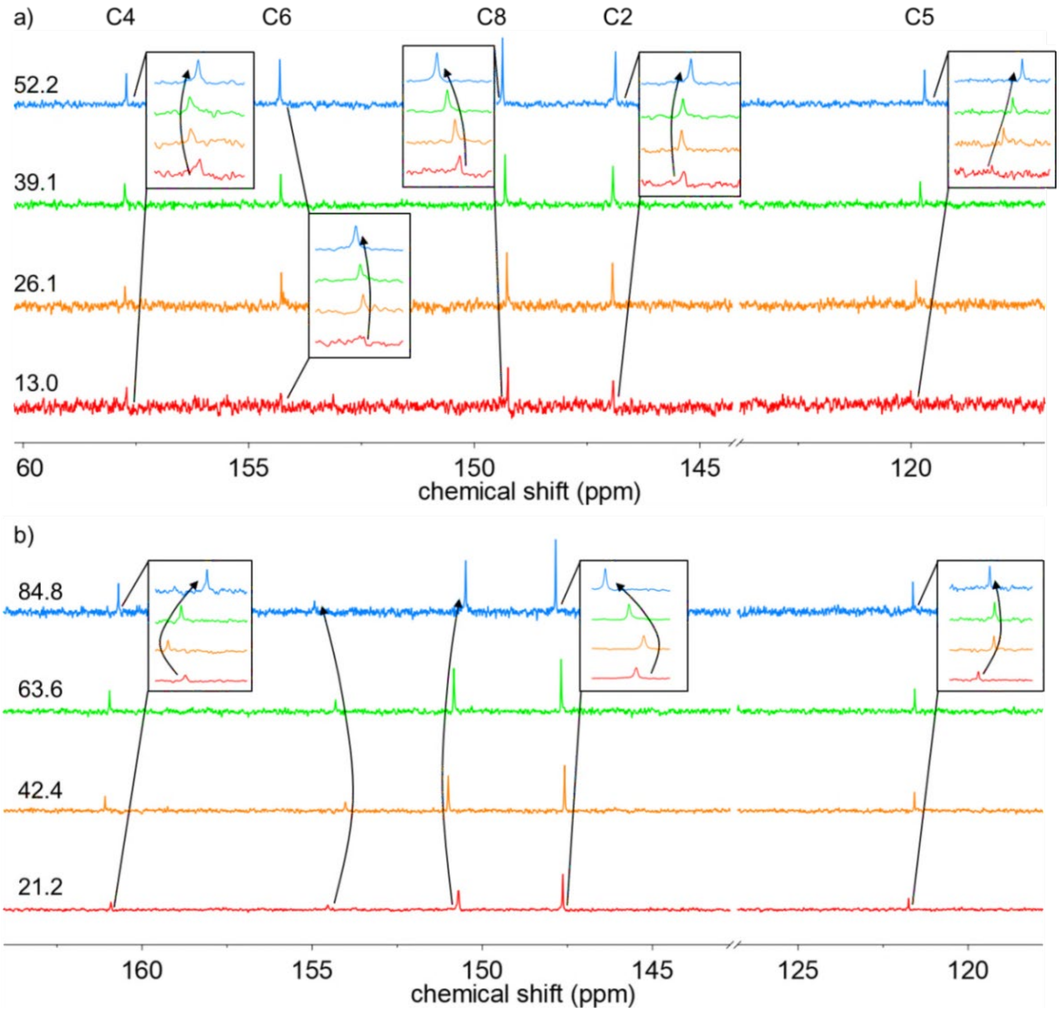

Figure S8 ^{13}C NMR spectra at various concentrations (indicated on the left, in mM) of a) Na-Ade and b) K-Ade, in anhydrous DMSO-d_6 at ambient temperature.

Temperature dependent studies were conducted over the range of concentrations used in order to investigate how increasing the temperature of the solution effects the interactions between molecules as the distance between the molecules is increased, reducing the intermolecular interactions between them.

The change in δ with temperature ($\Delta\delta_T = \delta_{T0} - \delta_{Tn}$, where δ_{T0} is the chemical shift of the reference point at the lowest temperature and δ_{Tn} of higher temperature) at the highest concentration used for each complex for the temperature range 300 K - 380 K is shown in Figure S9 (the same trends are observed at all concentrations used). For both cations, the temperature effect on the purine protons in the absence and presence of the crown ether is similar. For both Na- and K-Ade complexes the C2–H proton is increasingly deshielded ($\Delta\delta < 0$) with increasing temperature as is the C8–H of K-Ade but shielding ($\Delta\delta > 0$) is observed in the case of the C8–H and NH_2 protons of Na-Ade. The deshielding of the C2–H of both the Na- and K-Ade complexes and the C8–H of K-Ade with an increase in temperature suggests an influence of the cation as the concentration dependent deshielding of these protons in Figure S6 is not reversed with increase in temperature that would have been expected with the dissociation of the aggregates.

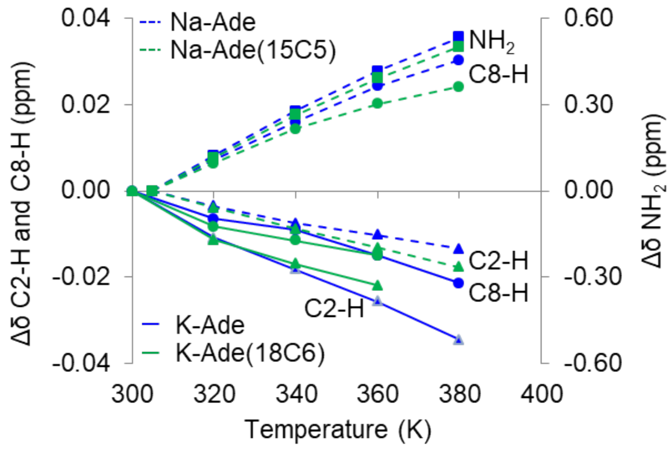

Figure S9: Change in ^{1}H NMR chemical shift of Na-Ade (52.2 mM), Na-Ade(15C5) (47.6 mM), K-Ade (84.8 mM) and K-Ade(18C6) (78.7 mM) at the indicated temperatures (K) in DMSO-d_6 for NH_2 (squares), C2–H (triangles) and C8–H (circles).

The change in the temperature coefficient for the purine protons of Na-Ade(15C5) and K-Ade(18C6) at various concentrations is shown in Figure S10. The presence of 15C5 ether resulted in a reduced temperature coefficient of the NH_2 protons in region 2 compared to Na-Ade indicating (i) shielding of these protons from the solvent or (ii) stronger hydrogen bonding involved in the aggregate formation as compared to that of the Na-Ade aggregates. The latter would suggest a difference in arrangement between the aggregated molecules of Na-Ade(15C5) and Na-Ade. For K-Ade, the presence of the 18C6 ether eliminates the difference in temperature coefficient between region 1 and 2 and there appears to be only a very slight concentration dependent change in the temperature coefficient.

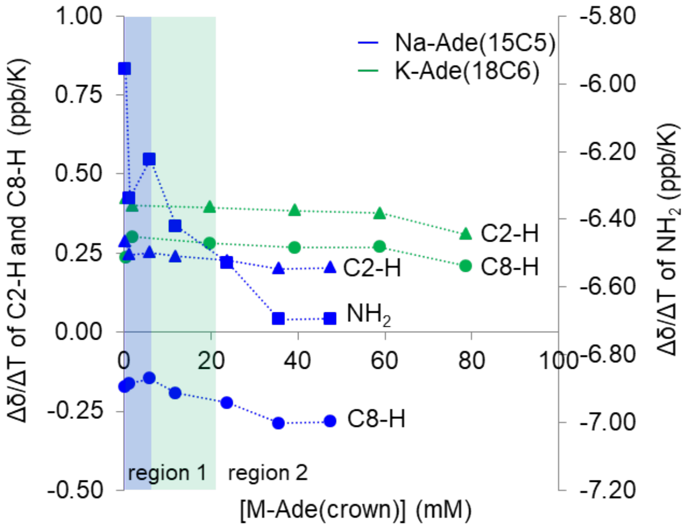

Figure S10: The change in temperature coefficients with concentration of ^{1}H chemical shifts for the Na-Ade(15C5) and K-Ade(18C6) in DMSO-d_6. NH_2 (squares), C8-H (circles) and C2-H (triangles).

Fitting of thermodynamic data

The expected sigmoidal curve generally obtained during aggregation studies is not obtained in the concentration or temperature dependent studies of Na-/K-Ade, Na-Ade(15C5) and K-Ade(18C6) in DMSO-d_6 due to change in chemical shift between region 1 and 2. The limit of dilution for ^{1}H NMR detection and saturation for solubility of the complexes added additional restrictions preventing the detection of monomer- and aggregate-only chemical shifts. Data from region 2 were used to obtain an estimate of aggregation for the ionic complexes. The limited data points for all complexes from the ^{1}H NMR study for region 1 prevented modelling of the aggregation occurring at these low concentrations. In region 2 increasing the concentration resulted in the δ for each purine proton to become deshielded as aggregates are formed. Increasing the temperature should yield less aggregates, resulting in shielding of the protons. However, the effect of temperature for a set of concentrations resulted in the δ being more deshielded, Figure S11.

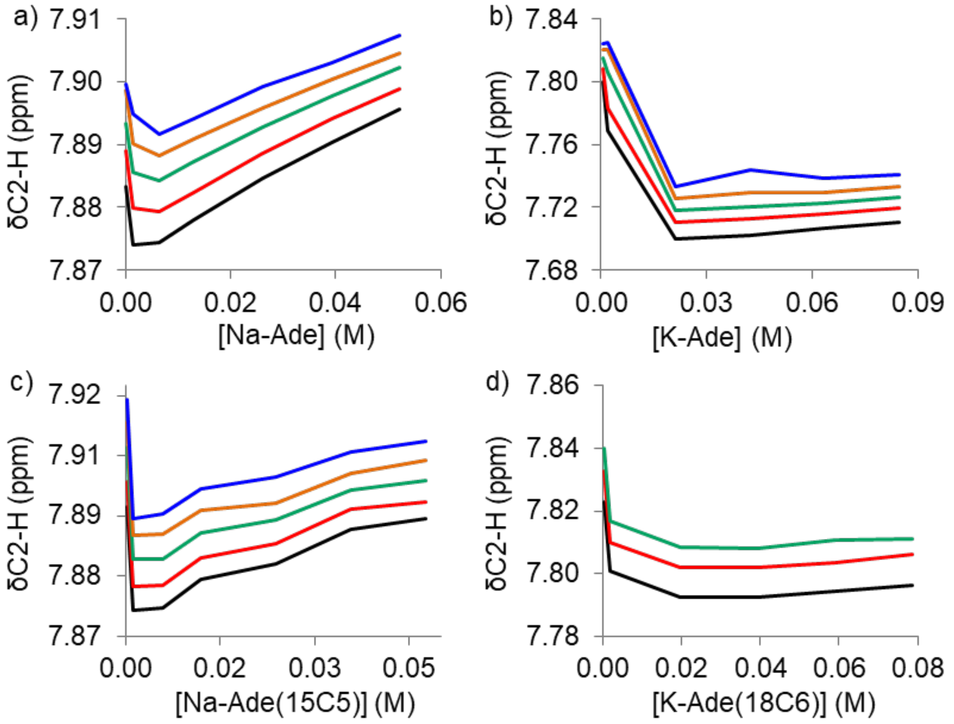

Figure S11: ^{1}H NMR chemical shift for the C2-H proton of a) Na-Ade, b) K-Ade, c) Na-Ade(15C5) and d) K-Ade(18C6) with an increase in concentration at 305 K (Na^+ complexes) or 300 K (K^+ complexes) (black line), 320 K (red line), 340 K (green line) 360 K (orange line) and 380 K (blue line).

The changes in chemical shifts of the purine protons with an increase in temperature were normalized, δ_N, in order to fit nonlinear NMR aggregation models. The more complex behavior of K-Ade(18C6) chemical shifts with an increase in temperature prevented model fitting, Figure S12d. The changes in chemical shift with concentration for each temperature (T) were normalized in order to obtain K_{eq} of self-aggregation in region 2 by the following method:

A normalization factor, *f*, is obtained for each T set in Figure S11,

$$f = \delta_{0_\mathrm{T}}/\delta_{0_{305}} \qquad (1)$$

where δ_{0_T} is the chemical shift at the reference concentration (lowest concentration at the beginning of the linear portion of phase 2) at temperature T (where T ≠ 305 or 300 K for the Na^+ and K^+ complexes, respectively, and $\delta_{0_{305}}$ is the chemical shift at the reference concentration at 305 or 300 K for the Na^+ and K^+ complexes, respectively. Reference concentrations are 6.52, 1.19, 21.2 and 19.7 mM for Na-Ade, Na-Ade(15C5), K-Ade and K-Ade(18C6), respectively.

The chemical shifts at concentrations larger than the reference concentration are normalized by the factor *f* obtained for each temperature:

$$\delta_\mathrm{N} = \delta_\mathrm{T}/f \qquad (2)$$

The normalized δ for the C2–H protons are shown in Figure S12.

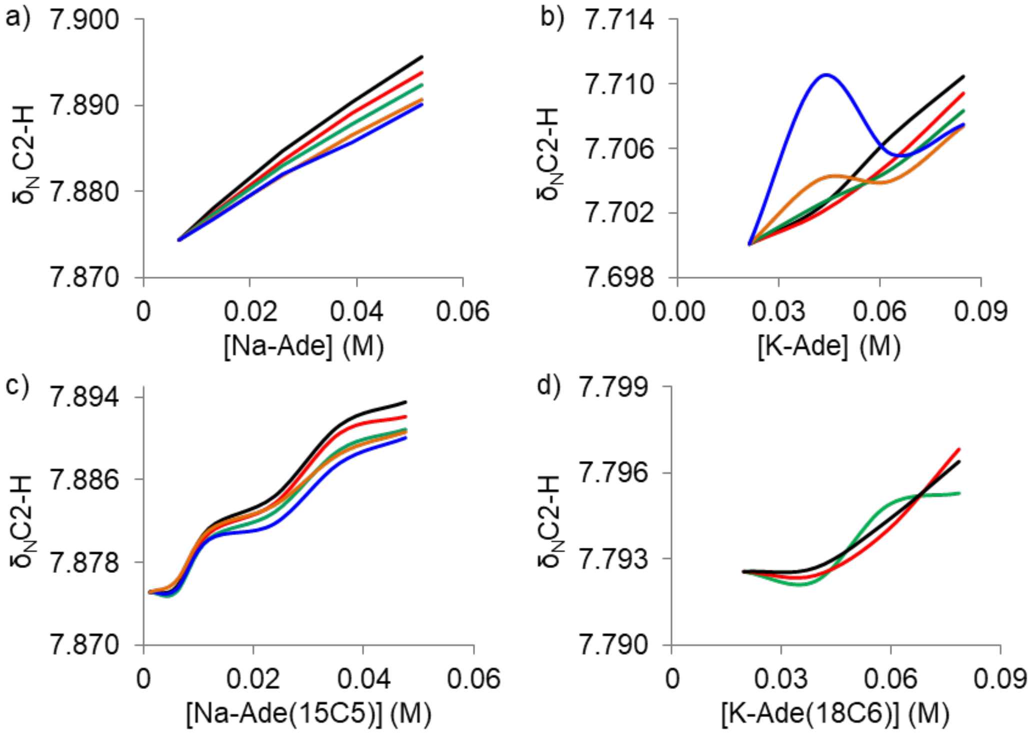

Figure S12: Normalized ^{1}H NMR chemical shifts for the C2-H proton of a) Na-Ade b) K-Ade, c) Na-Ade(15C5) and d) K-Ade(18C6) at black – 305 K (Na^+ complexes)/300 K (K^+ complexes), red – 320 K, green – 340 K, orange – 360 K, blue – 380 K.

In Figure S12b, there is a dramatic change in behavior of the K-Ade system at temperatures 360 and 380 K, indicating a change between different species in solution. This effect is not observed in the presence of the 18C6, Figure S12d.

Self-aggregation studies: The ^{1}H NMR spectra were recorded for a series of concentrations at 5 different temperatures (300/305, 320, 340, 360 and 380 K). Chemical shifts of ^{1}H NMR spectra were calibrated using residual non-deuterated solvent peaks of DMSO-d_6 according to temperature adjustments reported by Hoffman and Becker.[6] All analyses were performed in triplicate. The experimental ^{1}H NMR data were normalized as described in the SI. Aggregation between monomers (A) is considered in terms of dimer ($\mathrm{A_2}$, eqn (3)), trimer ($\mathrm{A_3}$, eqn (4)) or *n*-mer ($\mathrm{A_n}$, eqn(5)) formation.

$$2\mathrm{A} \rightleftharpoons \mathrm{A_2} \qquad K_2 = \frac{[\mathrm{A_2}]}{[\mathrm{A}]^2} \qquad (3)$$

$$3\mathrm{A} \rightleftharpoons \mathrm{A_3} \qquad K_3 = \frac{[\mathrm{A_3}]}{[\mathrm{A}]^3} \qquad (4)$$

$$\mathrm{nA} \rightleftharpoons \mathrm{A_n} \qquad K_n = \frac{[\mathrm{A_n}]}{[\mathrm{A}]^\mathrm{n}} \qquad (5)$$

To quantitatively analyse and interpret thermodynamic and kinetic data of aggregation, two mathematical models are used, the isodesmic[7] (eqn (6)) and trimer model,[8] eqn (7):

$$\delta_N = \delta_m + (\delta_a - \delta_m)\left(1 + \frac{1-\sqrt{4K_{agg}C_t+1}}{2K_{agg}C_t}\right) \qquad (6)$$

where $K_{agg} = K_2 = K_3 = K_n$,

$$\delta_N = \delta_m + 3C_t^2K_3(\delta_t - \delta_m) \qquad (7)$$

where $K_3 = \frac{[\mathrm{A_3}]}{[\mathrm{A}]^3}$

and where δ_N is the normalized observed chemical shift, δ_m and δ_a (or δ_t) is the chemical shifts of the monomer and aggregate (or trimer) forms, K_{agg} and K_3 are the aggregate and trimer formation constants and C_t is the total concentration of the ionic compound. The isodesmic model is the simplest aggregation model used to study the aggregation of small molecules. The trimer model was used when poor fit of the isodesmic model was obtained for K-Ade. The models were used to fit the normalized observed chemical shifts. Nonlinear regression analysis for the above models were fitted in OrginPro 8.5 to obtain K_{agg}, K_3, δ_m, δ_a, and δ_t for each proton, i.e. C2–H, C8–H and NH_2. The average over the three protons was used to draw the van't Hoff plot of $\ln K$ (where K is K_{agg} or K_3) vs $1/\mathrm{T}$ and the trend line was fitted using linear regression in OrginPro 8.5. The thermodynamic data was obtained from the slope ($\mathrm{slope} = -\Delta\mathrm{H}/\mathrm{R}$) and the intercept ($\mathrm{intercept} = \Delta\mathrm{S}/\mathrm{R}$) and ΔG using eqn (8)

$$\Delta G = \Delta H - T\Delta S \qquad (8)$$

The same procedure described above was performed for the guest-host model for the complexation of Na-Ade and 15C5. The concentration dependent ^{1}H NMR data of Na-

Ade(15C5) at the various temperatures were fitted using nonlinear regression to the guest-host model, assuming 1:1 complexation, eqn (9), in OriginPro 8.5.

$$\delta_N = \frac{\delta_0 + \delta_{\mathrm{AB}} K_1 \mathrm{C}_{\mathrm{Na}^+}}{1 + K_1 \mathrm{C}_{\mathrm{Na}^+}} \qquad (9)$$

where δ_0 and δ_{AB} is the chemical shift of the uncomplexed and complexed 15C5, K_1 is the association constant of 1:1 complexation between 15C5 and Na^+ and $\mathrm{C}_{\mathrm{Na}^+}$ is the concentration of Na^+.

Fitting of the isodesmic and trimer model to Na-Ade, Na-Ade(15C5) and K-Ade is shown in Figure S13, and the K_{agg} and K_3 at a given temperature for the purine protons are given in Table S1.

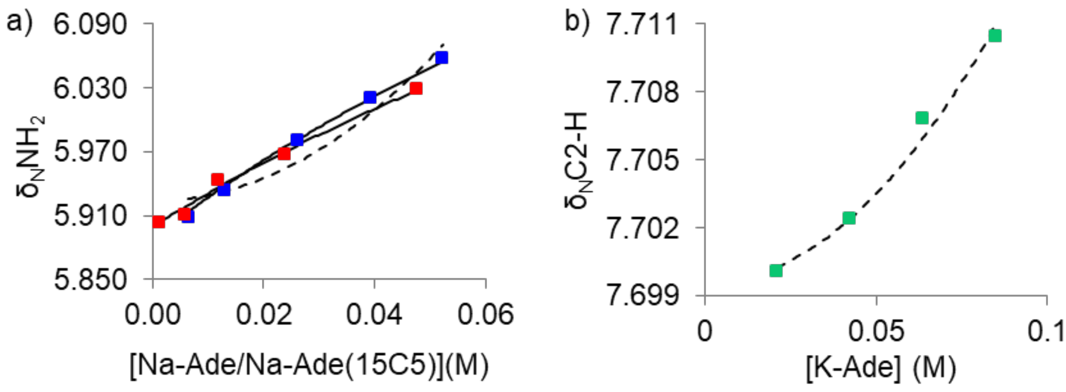

Figure S13: Fitting of the isodesmic (solid line) and trimer (dashed line) model to a) Na-Ade (blue squares) and Na-Ade(15C5) (red squares) for the NH_2 peaks and b) K-Ade (green squares) for the C2–H peak. Due to the extremely poor fit of the isodesmic model to K-Ade, data is not shown.

Table S1: K_{agg} constants obtained for the isodesmic model for Na-Ade and Na-Ade(15C5) and the K_3 for the trimer model for K-Ade at the indicated temperature range in DMSO-d_6 as solvent

	Na-Ade			Na-Ade(15C5)			K-Ade	
				K_{agg} (M^{-1})			K_3 (M^{-2})	
δ_m	5.89	7.87	7.62	5.90	7.87	7.63	7.40	7.70
δ_a	7.34	8.12	8.07	7.18	8.02	7.95	8.60	8.55
	NH2	C2–H	C8–H	NH2	C2–H	C8–H	C2–H	C8–H
305 K[a]	2.82	2.30	2.20	2.59	3.50	2.79	0.61	0.60
320 K	2.61	2.10	1.93	2.32	3.19	2.43	0.53	0.54
340 K	2.33	1.96	1.76	2.00	2.91	2.06	0.49	0.50
360 K	2.10	1.78	1.55	1.94	2.70	1.91		
380 K	1.97	1.72	1.44	2.27	3.00	2.04		

[a] 300 K for K-Ade

The average of the K_{agg} and K_3 at a given temperature for the purine protons in Table S1 were used to for the van't Hoff plot, Figure S15.

The 1:1 complexation between 15C5 and Na^+ was modelled using the concentration dependent change in CH_2 chemical shift of the 15C5 for the temperature range 305-380 K:

$$\delta_{obs} = \frac{\delta_0 + \delta_{AB} K_1 [C_{Na^+}]}{1 + K_1 [C_{Na^+}]} \quad (10)$$

where δ_0 and δ_{AB} is the chemical shift of the uncomplexed and complexed 15C5, K_1 is the association constant of 1:1 complexation between 15C5 and Na+ and C_{Na^+} is the concentration of Na^+. The fitted 1:1 model is shown in Figure S14 and association constants in Table S2.

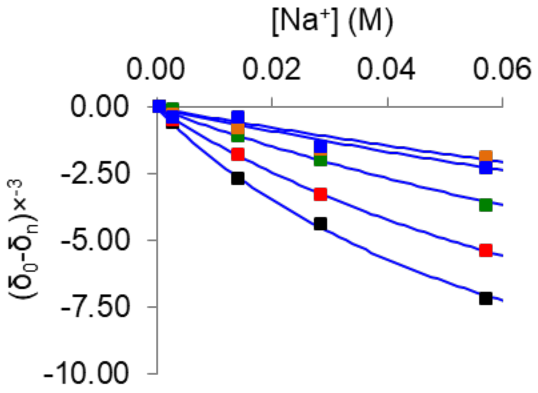

Figure S14: Fitting of the 1:1 model for the complexation between 15C5 and Na^+ at 305 K (black), 320 K (red), 340 K (green), 360 K (orange) and 380 K (blue)

Table S2: Association constant K_1 for the 1:1 complexation of 15C5 with Na^+ in DMSO in the presence of Ade^-

Temperature (K)	K_1 (M^{-1})
δ_0	–0.04954
δ_C	–15.86
305	27.9
320	18.0
340	10.0
360	5.78
380	4.90

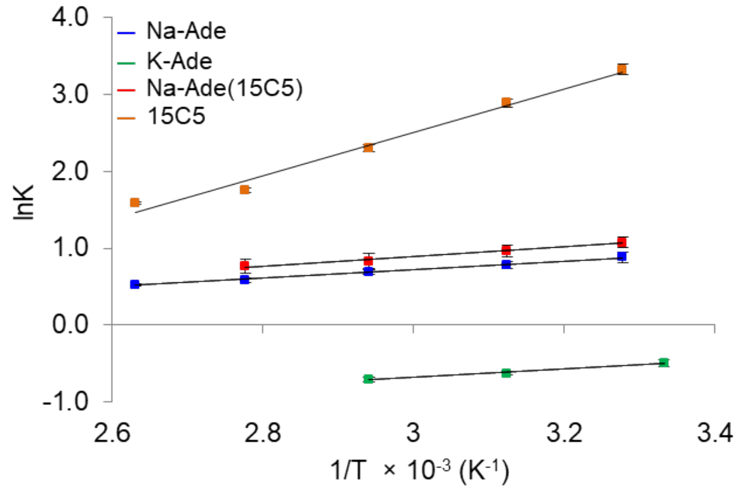

Figure S15: Van't Hoff plot of the equilibrium constant for the self-aggregation of Na-Ade, K-Ade and Na-Ade(15C5) and the 1:1 complexation between 15C5 ether and Na^+ in DMSO-d_6 as solvent.

Crystal structures of deprotonated adenine in the presence of sodium and potassium counter ion

Table S3: Structural information of the XRD crystal structure of Na-Ade, K-Ade and K-Ade(18C6) obtained in DMSO-d_6

Na-Ade	
DB05_3	Deposition Number 2055271
Space group	$P2_1/n$
Cell lengths	a 6.7911(2) b 9.1288(2) c 17.0758(4)
Cell angles	α 90 β 98.880(2) γ 90
Cell volume	1045.92
Z,Z'	Z: 4 Z': 0
R-factor	3.69
K-Ade	
DB02	-
Space group	$P\ 2_1/n$
Cell lengths	a 6.7960(6) b 9.1286(8) c 17.0547(15)
Cell angles	α 90 β 98.764(8) γ 90
Cell volume	1045.69
Z,Z'	Z: 4 Z': 0
R-factor	12.36
K-Ade(18C6)	Morphology 1 (main text)
cu_DB05_0m_a	Deposition Number 2055272
Space group	$P2_1/c$
Cell lengths	a 19.6287(9) b 8.5394(4) c 26.1208(12)
Cell angles	α 90 β 93.425(2) γ 90
Cell volume	4370.48
Z,Z'	Z: 8 Z': 0
R-factor	8.47

K-Ade(18C6)	Morphology 2
DB04	Deposition Number 2055270
Space group	P 2_1/m
Cell lengths	a 7.9183(4) b 16.2548(4) c 8.8051(3)
Cell angles	α 90 β 111.961(5) γ 90
Cell volume	1051.07
Z,Z'	Z: 2 Z': 0
R-factor	5.78

Table S4: Selected interatomic distances [Å] and dihedral angles [degrees] in the crystal structures of Na-Ade (M = Na) and K-Ade(18C6) (M = K)

Compound	Na-Ade[a]	K-Ade(18C6)[b]
Interatomic distances		
N1-M	2.517	
N3-M	3.232	2.915(2.910)
N9-M	2.442	2.945(2.924)
N7-M	2.453	
H12-N3	2.248	
H11-N7	2.465	2.217(2.191)
H12-N1	-	2.262(2.160)
d		0.924(0.928)
Covalently bonded atoms of the adeninate anion		
N1-C2	1.351	1.345 (1.339)
C2-N3	1.317	1.320
N3-C4	1.367	1.341 (1.348)
C4-C5	1.390	1.401
C4-N9	1.366	1.369
C5-C6	1.402	1.393 (1.408)
C5-N7	1.388	1.391 (1.365)
C6-N1	1.357	1.358 (1.365)
C6-N10	1.338	1.336
N7-C8	1.336	1.341 (1.334)
C8-N9	1.349	1.344(1.361)
Solvent and 18C6 interactions		
M-O (DMSO)	2.333	-
M-O (18C6)	-	2.789-3.023 (2.830-3.021)
Dihedral angles		
H11-N10-C6-N1	0.00	-11.27(13.22)
H12-N10-C6-C5	1.16	-18.66(5.86)
α (N3C4N9M)	−17.67	-0.09(2.34)
β (H13C2N1M)	17.25	-
γ (H14C8N7M)	−4.13	-

[a] Figure 2a [b] Figure 2b in main text

The XRD crystal structure obtained for Na-Ade(15C5) resulted in the same geometric parameters as those obtained for Na-Ade and therefore the data is not given.

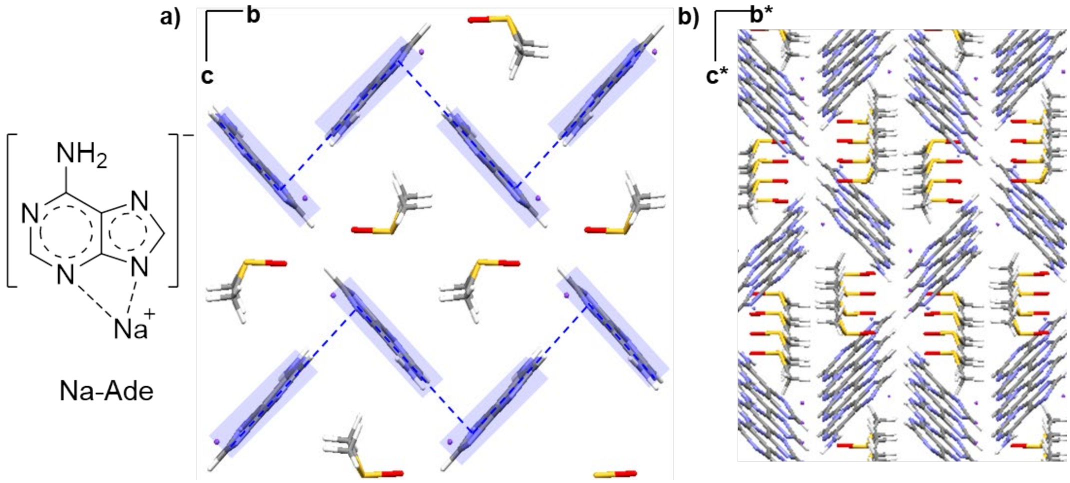

Figure S16: The molecular formula of Na-Ade and XRD the zigzag packing morphology of crystals with included solvent molecules obtained from DMSO-d_6 solutions, viewed along the a) a axis and b) reciprocal a axis.

The crystal structure of K-Ade had a poor R-factor (12.36). The R-factor reveals that the structure has not fully converged. However, this should not affect the unit cell and the arrangement of the ions within the cell, and we expect the connectivity between the ions is qualitatively correct. The structure can still be used for connectivity purposes to compare with the crystal structure of Na-Ade. The crystal packing of K-Ade, Figure S17, displays a zigzag packing morphology.

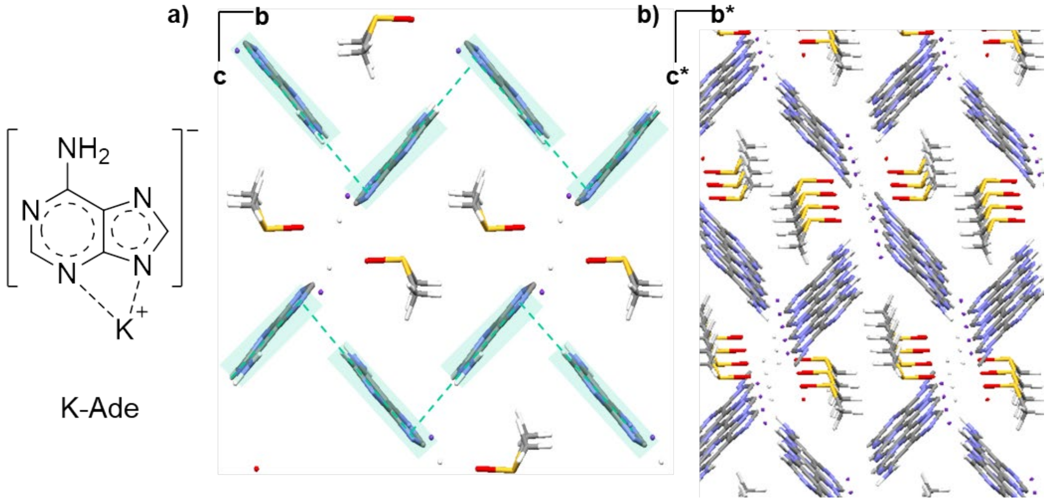

Figure S17: The molecular formula of K-Ade and XRD the zigzag packing morphology of crystals with included solvent molecules obtained from DMSO-d_6 solutions, viewed along the a) a axis and b) reciprocal a axis.

A closer look at the crystal packing shows that each K^+ counter ion is coordinated to three adeninate anion molecules via the N1-, N7- and N9-atoms of three purine rings of the adeninate anions (Figure S18).

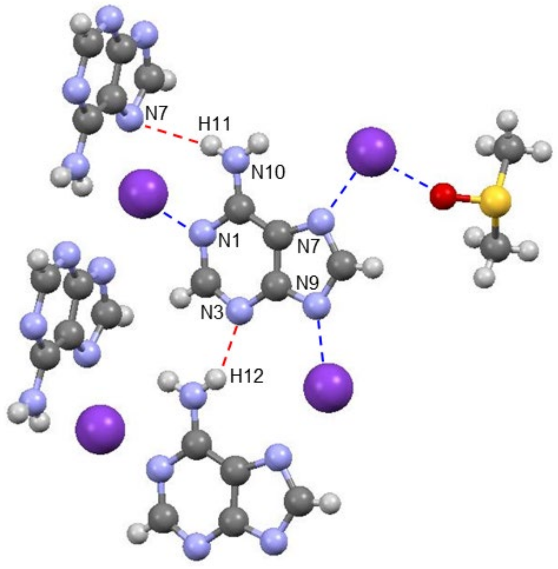

Figure S18: Crystal structure obtained from K-Ade showing one counter ion complexed with three adenine molecules and one adenine complexes with three counter ions.

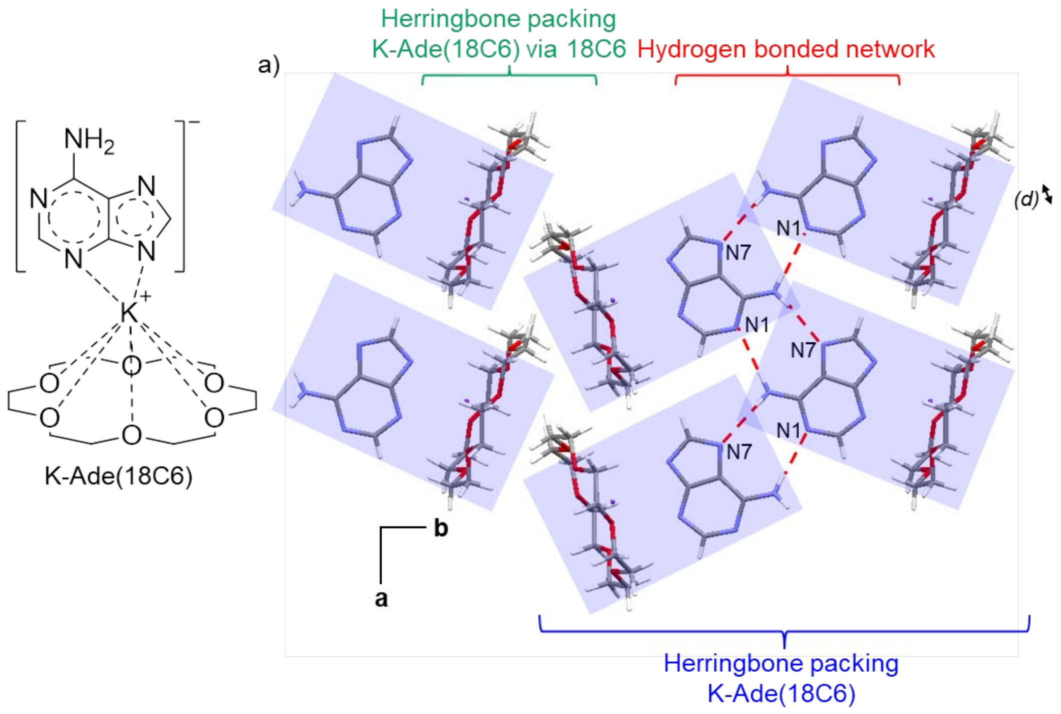

Figure S19: Molecular structure of K-Ade(18C6) and a): Herringbone (blue) XRD packing morphology of the K-Ade(18C6) crystal obtained from DMSO-d_6 solutions viewed along the c axis

The second crystal morphology obtained from the K-Ade(18C6) displayed positional disorder in the system, whereby 50% of the adeninate anion molecule occupies a different position, hence resulting in the disordered adeninate anion structure. Each adenine molecule has a potassium atom complexed to the N9 and N3 nitrogen atoms of the purine ring system and the 18-crown-6. The packing lacked the hydrogen-bonded network between the adenyl moieties observed in the first morphology (Figure S19), and instead displayed a brick wall pattern along the a-axis (Figure S19a) with each K-Ade(18C6) unit inverted relative to its neighbor and a slipped stacked packing along the b axis (Figure S20b).

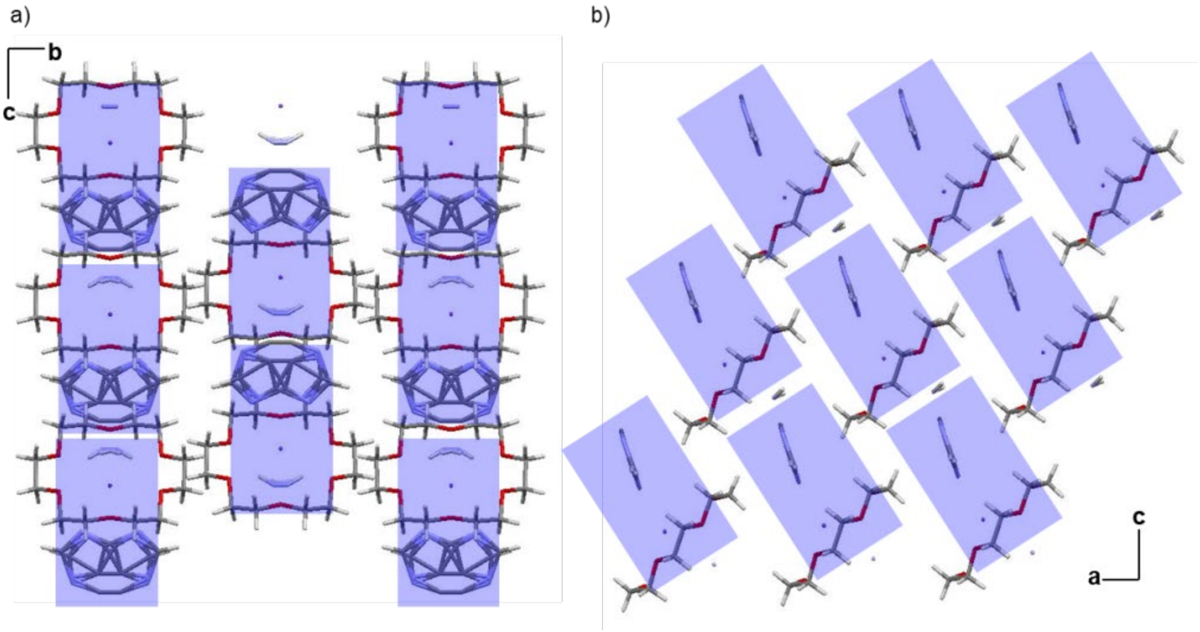

Figure S20: Packing of the second morphology of the adenine-potassium 18-crown-6 along the a) a and b) b axis.

Absorption and emission spectra of adenine-Na, adenine-K, adenine-Na 15-crown-5 and adenine-K 18-crown-6

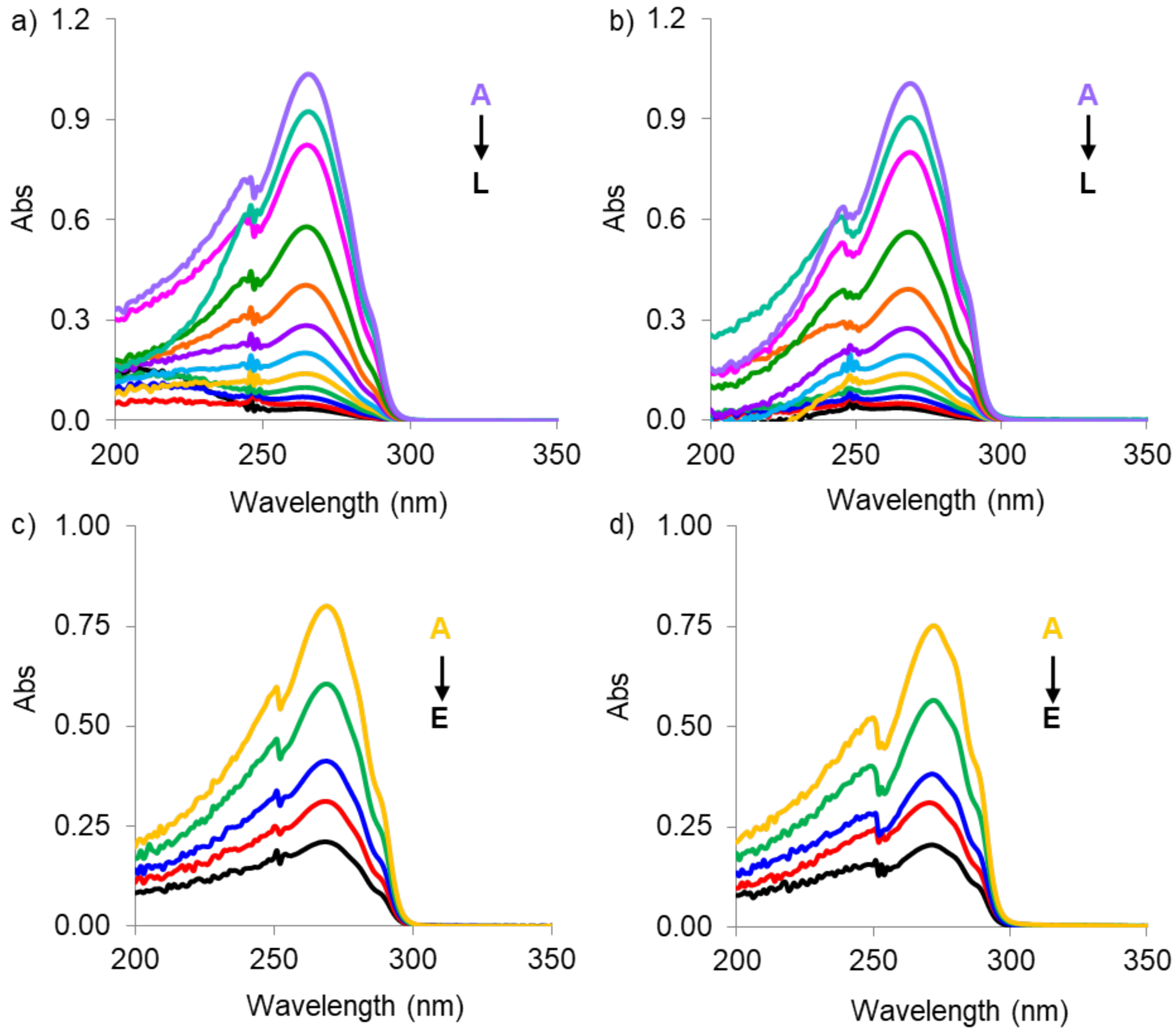

Figure S21: The concentration dependent UV absorption spectra of a) Na-Ade (A to H: 1.00 to 0.0323 mM) and b) K-Ade in DMSO as solvent (A to L: 1.00 to 0.0323 mM). Refer to Experimental details for UV spectroscopy studies for concentrations A to H/L. c) Na-Ade (A to E: 0.20 to 0.05 mM) and d) K-Ade (A to E: 0.20 to 0.05 mM) in anhydrous DMSO as solvent. A: 0.20, B: 0.15, C: 0.10, D: 0.075, E: 0.05 mM

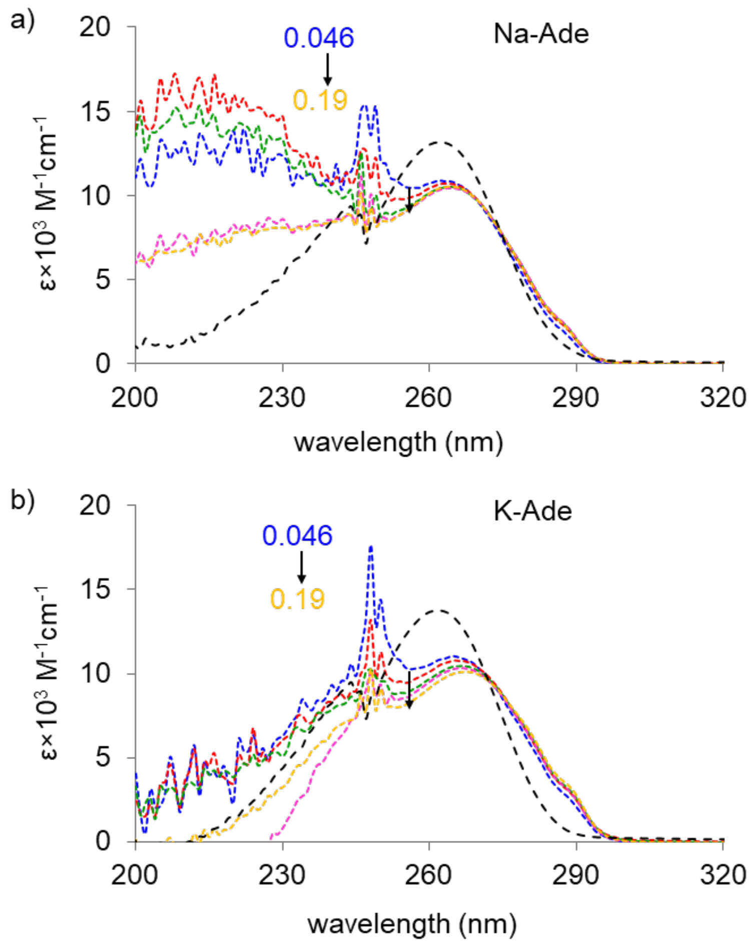

Figure S22: Concentration dependent UV absorption profile of a) Na-Ade and b) K-Ade in DMSO/H2O for the concentration range (0.046 – blue, 0.066 – red, 0.094 – green, 0.13 – pink, 0.19 – yellow, in mM).

The UV absorbance of KH in DMSO was run, Figure S22, in order to check the contribution of the hydride or free K^+ ion to the above K-Ade absorbance spectra. Solvated metal atoms do not have molecular vibrational states and hence display narrow absorption bands. These bands are significantly less prominent in anhydrous DMSO due to fewer free ions, and are absent in the presence of the 15C5 and 18C6 crown ether. In the Na-Ade DMSO UV spectra, a broad band lying below 235 nm is observed and is largely suppressed in anhydrous DMSO and in the presence of 15C5.

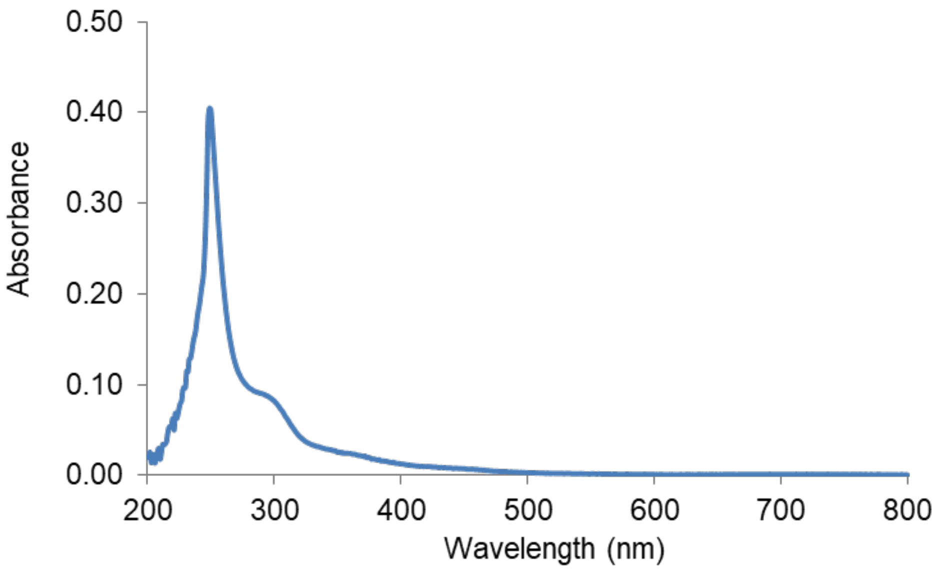

Figure S23: UV absorbance profile of KH (60.5 mM) in DMSO (no adenine present)

References:

[1] Noland W. E. *Organic Syntheses, Coll.* Vol. 6, p.301 (1988); Vol. 57, p.30 (1977)

[2] Rigaku Oxford Diffraction, CrysAlisPro Software system, (2018).

[3] APEX3 (including SAINT and SADABS), Bruker AXS Inc., Madison, WI, 2016.

[4] Sheldrick, G. M. SHELXT – Integrated space-group and crystal structure determination, *Acta Crystallogr., Sect. A: Found. Adv*. 2015, 71, 3–8.

[5] Sheldrick, G. M. Crystal structure refinement with SHELXL, *Acta Crystallogr., Sect. C: Struct. Chem*. 2015, 71, 3–8.

[6] Hoffman, R. E.; Becker, E. D. *J. Magn. Reson*. 2005, 176 (1), 87–98.

[7] Martin, R. B. *Chem. Rev.* 1996, 96 (8), 3043–3064.

[8] Formisano, S.; Johnson, M. L.; Edelhoch, H. *Proc. Natl. Acad. Sci.* 1977, 74 (8), 3340–3344.
